# Evaluation of cellular activity in response to sleep deprivation by a comprehensive analysis of the whole mouse brain

**DOI:** 10.3389/fnins.2023.1252689

**Published:** 2023-10-19

**Authors:** Takuya Urushihata, Mio Goto, Keiko Kabetani, Mai Kiyozuka, Shiho Maruyama, Shogo Tsuji, Hirobumi Tada, Akiko Satoh

**Affiliations:** ^1^Department of Integrative Physiology, Institute of Development, Aging and Cancer, Tohoku University, Sendai, Japan; ^2^Department of Integrative Physiology, National Center for Geriatrics and Gerontology, Obu, Japan; ^3^Department of Nutrition, Faculty of Wellness, Shigakkan University, Obu, Japan; ^4^Department of Physiology, Yokohama City University Graduate School of Medicine, Yokohama, Japan

**Keywords:** sleep deprivation, cFos, TRAP2, whole brain, neural circuits

## Abstract

Sleep deprivation (SD) causes several adverse functional outcomes, and understanding the associated processes can improve quality of life. Although the effects of SD on neuronal activity in several brain regions have been identified, a comprehensive evaluation of the whole brain is still lacking. Hence, we performed SD using two different methods, gentle handling and a dedicated chamber, in targeted recombination in active populations 2 (TRAP2) mice crossed with Rosa-ZsGreen reporter mice and visualized cellular activity in the whole brain. Using the semi-automated post-imaging analysis tool Slice Histology Alignment, Registration, and Cell Quantification (SHARCQ), the number of activated cells was quantified. From the analysis of 14 brain regions, cellular activity was significantly increased in the olfactory areas and decreased in the medulla by the two SD methods. From the analysis of the further subdivided 348 regions, cellular activity was significantly increased in the vascular organ of the lamina terminalis, lateral hypothalamic area, parabigeminal nucleus, ventral tegmental area, and magnocellular reticular nucleus, and decreased in the anterior part of the basolateral amygdalar nucleus, nucleus accumbens, septohippocampal nucleus, reticular nucleus of the thalamus, preoptic part of the periventricular hypothalamic nucleus, ventromedial preoptic nucleus, rostral linear nucleus raphe, facial motor nucleus, vestibular nuclei, and some fiber tracts (oculomotor nerve, genu of corpus callosum, and rubrospinal tract) by the two SD methods. Two subdivided regions of the striatum (caudoputamen and other striatum), epithalamus, vascular organ of the lamina terminalis, anteroventral preoptic nucleus, superior colliculus optic layer, medial terminal nucleus of the accessory optic tract, pontine gray, and fiber tracts (medial lemniscus, columns of the fornix, brachium of the inferior colliculus, and mammillary peduncle) were differentially affected by the two SD methods. Most brain regions detected from these analyses have been reported to be involved in regulating sleep/wake regulatory circuits. Moreover, the results from the connectivity analysis indicated that the connectivity of cellular activity among brain regions was altered by SD. Together, such a comprehensive analysis of the whole brain is useful for understanding the mechanisms by which SD and/or sleep disruption affects brain function.

## 1. Introduction

Inadequate quantity or quality of sleep is linked to various adverse health outcomes. For instance, acute sleep deprivation (SD) results in poor emotional ability and cognition (Goel et al., 2013; Hudson et al., 2020). Chronic SD affects energy metabolism, presumably leading to obesity, diabetes, and cardiovascular disease (Watanabe et al., 2010; Grandner et al., 2013, 2015; Kohansieh and Makaryus, 2015; Shan et al., 2015). Moreover, comorbid sleep disruption is often observed in major neurological and psychiatric disorders, including schizophrenia, anxiety disorders, addiction disorders, and Alzheimer’s disease (Krystal, 2012; Bishir et al., 2020; Wang and Holtzman, 2020). Thus, understanding the detailed mechanisms by which acute/chronic SD and sleep disruption affect brain function could improve quality of life.

Previous studies have reported that cellular activity is altered in response to SD in rodent models (O’Hara et al., 1993; Cirelli et al., 1995; Semba et al., 2001; Terao et al., 2003; Jha et al., 2017; Sardi et al., 2018; Montes-Rodríguez et al., 2019) and humans (Chee et al., 2008; De Havas et al., 2012; Ma et al., 2015). However, because most rodent studies have focused on only each brain area such as the cerebral cortex, hippocampus, striatum, pallidum, thalamus, hypothalamus, midbrain, pons, and cerebellum (Merchant-Nancy et al., 1992; O’Hara et al., 1993; Cirelli et al., 1995; Semba et al., 2001; Terao et al., 2003; Jha et al., 2017; Sardi et al., 2018; Montes-Rodríguez et al., 2019), several critical brain regions and/or functional characteristics in response to SD have not been uncovered. Additionally, the technique for promoting SD is valid for each study, particularly a chronic SD study (Cai et al., 2022). Therefore, a comparison between different techniques would be ideal to further elucidate the effect of SD.

It is important to evaluate cellular activity in the whole brain during SD using a comprehensive method. For instance, functional magnetic resonance imaging (fMRI) is often used to examine whole-brain functions. However, fMRI can only measure time series of data with a relatively low resolution, which makes it difficult to precisely evaluate brain activity in subdivided brain regions (Markicevic et al., 2021). A direct count of activated cells using labeling techniques is one of the best ways to evaluate SD-induced changes in cellular activity in subdivided brain regions. The recent development of targeted recombination in active population (TRAP) mice provides a great methodology for evaluating activated cells during a specific period (Guenthner et al., 2013; Allen et al., 2017; DeNardo et al., 2019). TRAP and its new version, TRAP2, allow inducible iCre recombination in cFos-expressing cells when 4-hydroxytamoxifen (4-OHT) is present. Importantly, cells labeled in TRAP2 mice consistent with endogenous cFos expression with high efficiency (Allen et al., 2017; DeNardo et al., 2019; Chen et al., 2020). These cFos-TRAPed cells can be visualized by crossing with reporter mice that can express fluorescence. Slice Histology Alignment, Registration, and Cell Quantification (SHARCQ), a post-imaging analysis tool, has recently been developed (Lauridsen et al., 2022). SHARCQ semi-automatically registers histological images of brain slices onto the mouse brain atlas and counts labeled cells for each brain region (Lauridsen et al., 2022). Given that the SHARCQ is designed for an unbiased approach to whole-brain mapping, the combination of the TRAP technique and SHARCQ allows us to comprehensively evaluate whole-brain activity at cell-level resolution.

Therefore, in this study, we examined 6-h SD using two different methods and evaluated cellular activity in the whole brain using TRAP2 mice and SHARCQ. We evaluated whether such a comprehensive analysis of the whole brain can highlight brain regions and their networks that are known or unknown to be involved in sleep/wake circuits.

## 2. Materials and methods

### 2.1. Mice

Fos^2A-iCreERT2^ (TRAP2) mice (030323, Jackson Laboratory) were crossed with Rosa26-ZsGreen mice (007906, Jackson Laboratory) to obtain double-heterozygous (TRAP2;Rosa26-ZsGreen) mice. Eight TRAP2;Rosa26-ZsGreen males weighing 30–40 g at 12 months of age were used in the experiment (two biological replicates for each group). Mice were housed under a 12/12-h light/dark cycle (lights on at 6 am and off at 6 pm) with free access to food and water. We employed only males for the current experiments to avoid estrus-related effects on sleep regulation (Schwierin et al., 1998; Tóth et al., 2020). All mouse experiments and procedures were approved by the Animal Care and Use Committee at National Center for Geriatrics and Gerontology.

### 2.2. Preparation of 4-hydroxytamoxifen

4-OHT (H6278, Sigma) was dissolved at 20 mg/mL in ethanol by shaking at 37°C for 15 min (aliquots were stored at −20°C for up to several weeks) (Allen et al., 2017). On the day of the experiment, 4-OHT was redissolved in ethanol by shaking at 37°C for 15 min with a 1:4 mixture of castor oil (259853, Sigma); sunflower seed oil (S5007, Sigma) was added to obtain a final concentration of 10 mg/mL. 4-OHT, and the ethanol was evaporated under vacuum centrifugation.

### 2.3. Sleep deprivation study

**Figure 1:**
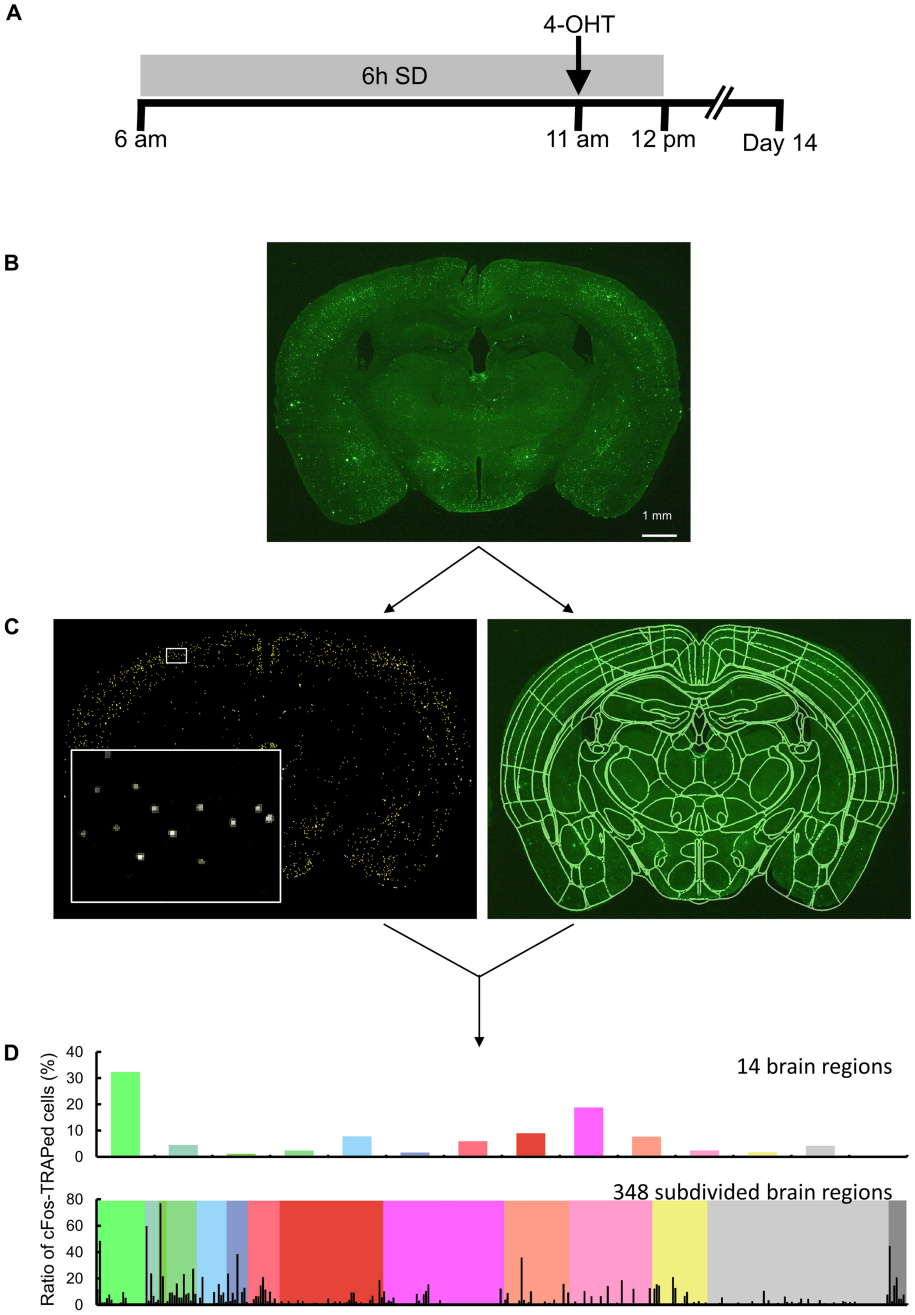
Overview of the study approach. **(A)** Schematic drawing of sleep deprivation (SD) and 4-OHT injection timelines. **(B)** Brain sections of TRAP2;Rosa26-ZsGreen mice, with or without SD, were measured by fluorescence imaging. Scale bar indicates 1 mm. (**C**, left) Following image preprocessing, cFos-TRAPed cells were labeled by thresholding, and their x and y coordinates were obtained using Image J. Each yellow ROI outlines a valid cFos-TRAPed cell, and the magnified view of the area enclosed by the white box is shown on the bottom left. (**C**, right) 3D segmentation of brain sections was performed by semi-automated registration (SHARCQ, Lauridsen et al., 2022) to the Allen brain atlas (Wang et al., 2020) using MATLAB. **(D)** The ratios of cFos-TRAPed cells in 14 brain regions were quantified and compared. For a total of 348 subdivided regions, the ratios of cFos-TRAPed cells in each of the 14 regions were obtained.

SD was performed using two methods: gentle handling and a dedicated chamber. For SD by gentle handling, mice were individually housed prior to the experiment. On the day of SD, mice were kept awake using a long Q-tip from 6 am to 12 pm by gently touching the mice (SD-H), as previously reported (Franken et al., 1991). Mice used for control manipulation (*ad libitum* sleep-H) were housed individually. For SD with chambers, two mice were habituated for 1 week in a dedicated chamber (Model 80391, Lafayette Instrument Company). On the day of SD, the mice were kept awake by a bar that automatically moved at the bottom of the chamber every minute from 6 am to 12 pm (SD-C). The bar was moved horizontally for 7.5 s during each sweep. Mice for control manipulation (*ad libitum* sleep-C) were housed in the same chamber throughout the experimental period. Five hours after the start of SD, 4-OHT was injected intraperitoneally at doses based on the weight of the mice (50 mg/kg BW). After SD, the mice were allowed to sleep. We used 4-OHT to label the activated cells in TRAP2;Rosa26-ZsGreen mice. 4-OHT initiates labeling more rapidly than tamoxifen, and labeling of activated cells occurs within a 6-h window around the 4-OHT injection (Figure 1A; Guenthner et al., 2013; Cazzulino et al., 2016; DeNardo et al., 2019).

### 2.4. Fluorescence imaging

Two weeks after the 4-OHT injection, the mice were anesthetized with isoflurane and perfused with PBS followed by 4% paraformaldehyde (PFA). The brains were fixed with 4% PFA overnight and placed in 30% sucrose until saturation. Thirty-micrometer cryosections were collected into PBS and stored in cryoprotectant at −20°C until further processing. The sections were mounted onto slides using antifade Vectashield (Vector Laboratories). Brain sections were imaged using a stereomicroscope equipped with a camera (M165 FC, Leica Microsystems GmbH). The excitation light (470/40 nm) was applied to the brain sections, and fluorescence was detected using a bandpass filter for GFP (525/50 mm) (Figure 1B). A single image plane consisted of 2,592 × 1,944 pixels, and the in-plane pixel size was 5.0 μm.

### 2.5. Image analysis

Image analysis was performed in the anterior–posterior (AP) range: +1.3 mm to −5.9 mm (total of 25 images). Areas of the isocortex, olfactory areas, hippocampal formation, and cortical subplate in the AP −3.2 to −4.7 mm range were excluded from analysis because the cerebral cortex was often lacking in this range during section preparation. ImageJ (NIH) was used for image preprocessing and detection of the x and y coordinates of the ZsGreen-expressing cells. The original image was median filtered (3 × 3 pixels) and subtracted from the background image created by the rolling ball algorithm (a radius of 5 pixels was used). The x and y coordinates of the centroid were obtained for each connected element with pixel intensity 20 or more and were defined as cFos-TRAPed cells (Figure 1C). The connected elements (less than 2 pixels) were excluded. Although it has been reported that more than 96% of cFos-TRAPed cells are neurons and the remaining <4% of cells include putative endothelial and glial cells (Guenthner et al., 2013), these were not distinguished in the present study. By using MATLAB (MathWorks), the images were semi-automatically registered to Allen Brain Atlas (Wang et al., 2020) based on the SHARCQ (Lauridsen et al., 2022; Figure 1C), and then the number of cFos-TRAPed cells were automatically counted in 14 brain regions or 348 subdivided brain regions (Figure 1D). The total number of cFos-TRAPed cells did not differ from each other among groups (29,621 ± 2,826) (Supplementary Table S1). Thus, the ratios of the number of cFos-TRAPed cells in each region to the total number of all brain sections (cFos-TRAPed ratio) were calculated and used for further analyses. For the subdivided brain regions, the ratios of the number of cFos-TRAPed cells in each subdivided region to the total number of brain sections in each one of the 14 brain regions were calculated.

### 2.6. Brain connectivity analysis

Connectivity among a total of 294 regions, excluding the regions with no cFos-TRAPed cell expression from the 348 subdivided regions, was estimated by calculating the covariance across mice (Wheeler et al., 2013). First, for comparing *ad libitum* sleep and SD, the data were combined into a group of sum_*ad libitum* sleep (*ad libitum* sleep-C and *ad libitum* sleep-H) or sum_SD (SD-C and SD-H). For comparison between groups H and C, the data of both conditions were combined as a group of sum_chamber (*ad libitum* sleep-C and SD-C) or sum_hand (ad libitum sleep-H and SD-H). Second, pairwise correlations among the 294 regions were estimated by computing Pearson correlation coefficients of the cFos-TRAPed ratio (total number of correlations was 43,071). Third, for each of the 14 regions, correlation coefficients were averaged to obtain the mean r. In addition, the positive mean r, which averaged only positive values; the negative mean r, which averaged only negative values; and the absolute mean r, which averaged the absolute values of the correlation coefficients, were also calculated.

### 2.7. Sleep analysis

Isoflurane-anesthetized mice were surgically implanted with stainless steel screw electrodes placed over the right frontal bone for reference and right parietal bone for active recording electroencephalogram (EEG). Furthermore, wire electrodes were implanted in the nuchal muscle for recording electromyogram (EMG) recording. After a three-day recovery period following surgery, the mice were recovered from surgery for 3 days and subsequently acclimatized to the recording cage for 3 weeks. Wireless EEG Logger (ELG-2, Bio research Center) was used for sleep analysis (Tsuji et al., 2023). Ten-second epochs of EEG/EMG signals were semiautomatically scored as wakefulness, non-rapid eye movement (NREM), or rapid eye movement (REM) sleep by visual examination using SleepSign (KISSEI COMTEC). The scores were blinded for groups during quantification.

### 2.8. Statistical analysis

Excel and GraphPad Prizm were used for data quantification and the generation of graphs. Statistical analyses were performed using the Statistics and Machine Learning Toolbox of MATLAB. Values were presented as mean with the value of each animal or mean ± standard error of the mean (SEM). Ratios of cFos-TRAPed cells were compared using a two-way ANOVA. The correlation coefficient among each region was tested using the two-sample Kolmogorov–Smirnov test. The percentages of wakefulness/NREM/REM sleep were determined using Student’s *t*-test. The null hypothesis was rejected when the *p*-value was less than 0.05.

## 3. Results

### 3.1. Both SD methods promoted predominant wakefulness

**Figure 2:**
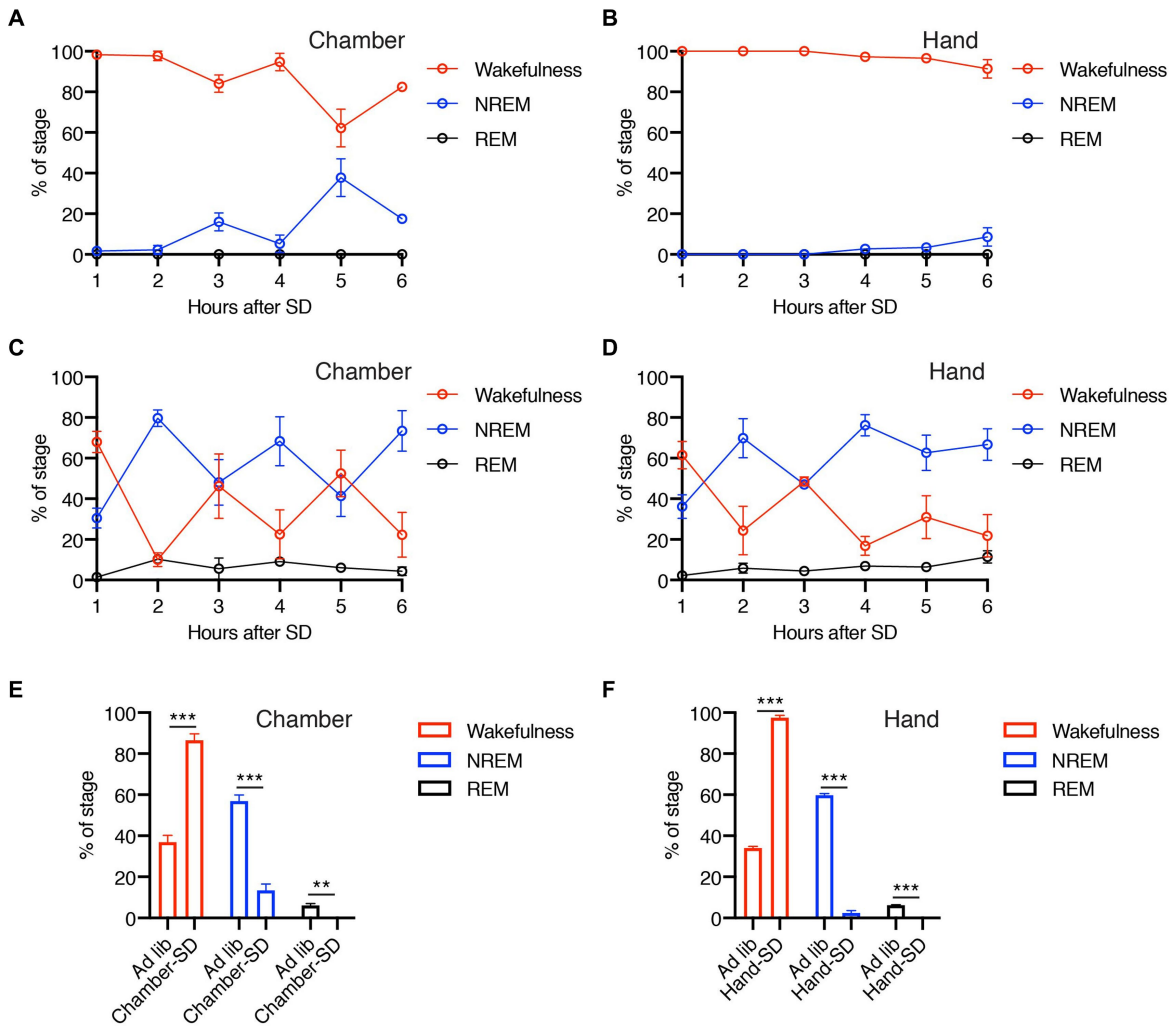
**(A–D)** Percentage of wakefulness and NREM and REM sleep during 6-h SD period using dedicated chamber **(A)** or gentle handling **(B)** and each *ad libitum* sleep **(C,D)**. **(E,F)** Total percentage of wakefulness and NREM and REM sleep during 6 h of SD or *ad libitum* sleep using dedicated chamber **(E)** or gentle handling **(F)**. Error bars indicate SEM (*N* = 3–4). **p < 0.01 and ***p < 0.001 for the difference between *ad libitum* sleep mice and SD mice by Student’s *t*-test.

In this study, we performed SD using two different methods, gentle handling and a dedicated chamber. Figure 2 demonstrates the efficacy of our methods during the 6-h SD period. The mice showed predominant wakefulness during SD by both SD-H and SD-C methods (Figures 2A,B), compared with baseline conditions (Figures 2C,D). The level of wakefulness obtained by SD-C and SD-H was significantly higher than that during *ad libitum* sleep, whereas the levels of NREM and REM sleep were significantly lower (Figures 2E,F). These results indicated that both our methods were effective in promoting a significant reduction in sleep duration.

### 3.2. Expression ratios of cFos-TRAPed cells in the olfactory area and medulla were altered by sleep deprivation

**Figure 3:**
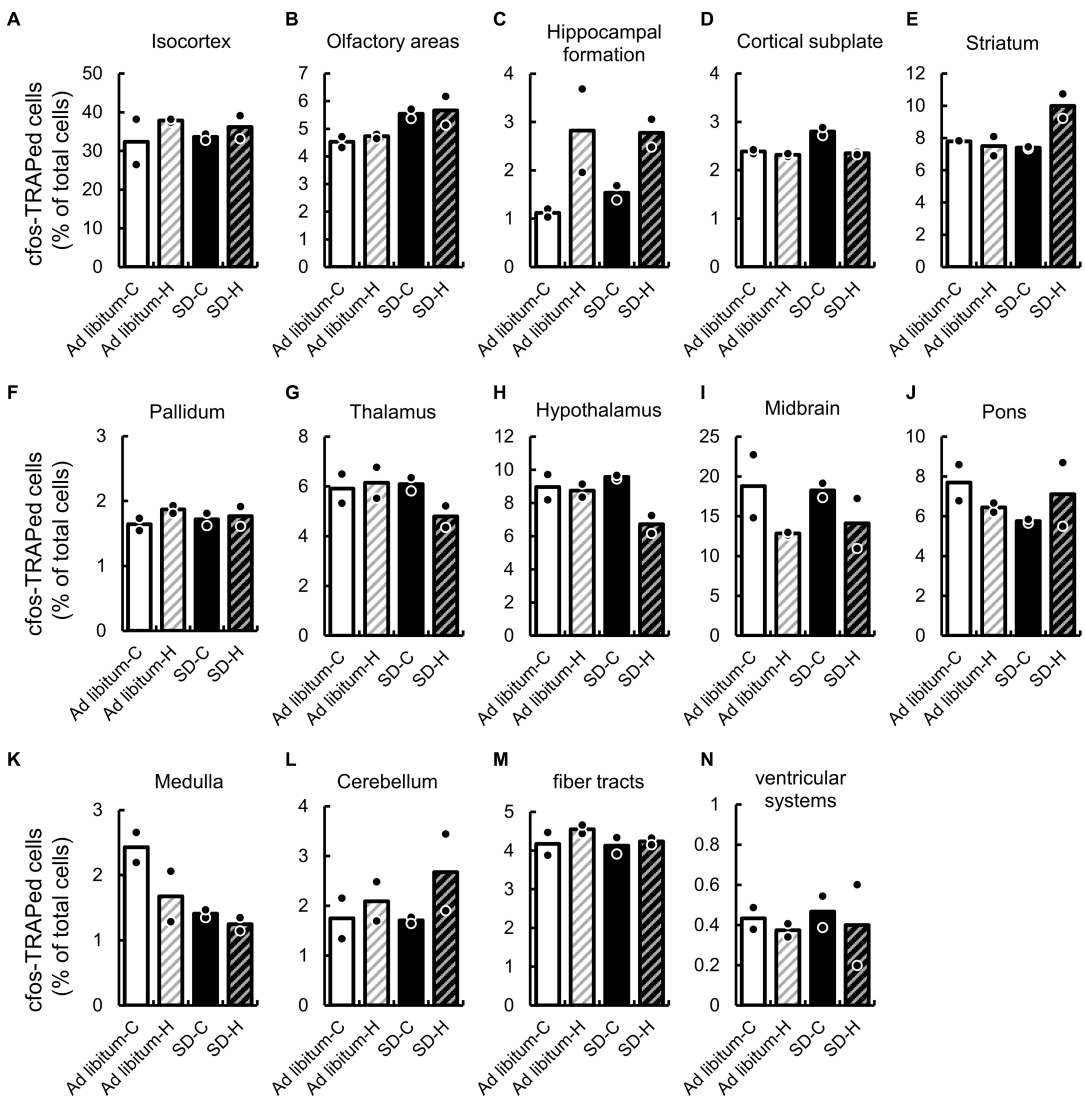
SD-induced changes of cFos-TRAPed cells ratio in 14 brain regions. The cFos-TRAPed cell ratios in 14 brain regions, **(A)** isocortex; **(B)** olfactory areas; **(C)**, hippocampal formation; **(D)** cortical subplate; **(E)** striatum; **(F)** pallidum; **(G)** thalamus; **(H)** hypothalamus; **(I)** midbrain; **(J)** pons; **(K)** medulla; **(L)** cerebellum; **(M)** fiber tracts; and **(N)** ventricular systems are shown. For each group, the plots and bars correspond to the value for each animal and the mean of the animals (*N* = 2, respectively). The values and two-way ANOVA results are shown in Supplementary Table S2.

We first compared the expression ratio of cFos-TRAPed cells between *ad libitum* sleep and SD in the 14 brain regions, which are defined by a broad regional classification of the brain (Lauridsen et al., 2022), including the isocortex, olfactory areas, hippocampal formation, cortical subplate, striatum, pallidum, thalamus, hypothalamus, midbrain, pons, medulla, cerebellum, fiber tracts, and ventricular systems (Figure 3 and Supplementary Table S2). After SD, the expression ratios of cFos-TRAPed cells were significantly higher in the olfactory areas (1.22 and 1.20-fold by SD-C and SD-H, respectively) and cortical subplate (1.17 and 1.01-fold by SD-C and SD-H, respectively), while they were lower in the medulla (0.58 and 0.74-fold by SD-C and SD-H, respectively) compared with *ad libitum* sleep (Figures 3B,D,K). There was a significant interaction between these two variables in the cortical subplate (Figure 3D and Supplementary Table S2). Thus, these results indicate that both types of SD methods have a common effect on cellular activity in the olfactory area and medulla among brain regions.

We also evaluated the expression ratio of cFos-TRAPed cells between groups H and C. The expression ratio of cFos-TRAPed cells in group H significantly differed from that in group C in the hippocampal formation (2.5 and 1.80-fold by *ad libitum* sleep-C and SD-C, respectively), cortical subplate (0.97 and 0.84-fold by *ad libitum* sleep-C and SD-C, respectively), and hypothalamus (0.98 and 0.70-fold by *ad libitum* sleep-C and SD-C, respectively) (Figures 3C,D,H). As mentioned previously, a significant interaction between the two variables was observed in the cortical subplate (Supplementary Table S2). These results indicate that groups H and C showed different effects on cellular activity in the hippocampal formation and hypothalamus, but not in the cortical subplate. Moreover, a significant interaction between the two variables was also observed in the striatum and cortical subplate (Figure 3E). It is suggested that cellular activity in the cortical subplate and striatum might differ between the two different SD methods.

### 3.3. Expression ratios of cFos-TRAPed cells in the several subdivided regions were altered by sleep deprivation

Concern existed that differences in the expression ratios of cFos-TRAPed cells at the 14 brain regions could be overestimated or underestimated (Figure 3 and Supplementary Table S2). Therefore, we compared the expression ratios of cFos-TRAPed cells obtained from the 348 subdivided regions for each of the 14 brain regions (depth 7 and 6 for the hypothalamus and the other 13 brain regions, respectively) (Table 1 and Supplementary Table S3). After SD, the expression ratios of cFos-TRAPed cells were significantly higher in the striatum (CP: 1.15 and 1.46-fold by SD-C and SD-H, respectively), hypothalamus (OV: 6.3-fold by SD-C; LHA: 1.16 and 1.19-fold by SD-C and SD-H, respectively), midbrain (PBG: not determined; VTA: 2.02 and 1.78-fold by SD-C and SD-H, respectively), and medulla (MARN: 1.41 and 2.36-fold by SD-C and SD-H, respectively) compared with *ad libitum* sleep (Table 1). In contrast, it was lower after SD in the cortical subplate (BLAa: 0.95 and 0.77-fold by SD-C and SD-H, respectively), striatum (ACB: 0.70 and 0.76; SH: not determined and 0.20-fold by SD-C and SD-H, respectively), thalamus (RT: 0.54 and 0.72-fold by SD-C and SD-H, respectively), hypothalamus (PVpo: 0.44 and 0.49; VMPO: 0.05 and 0.66-fold by SD-C and SD-H, respectively), midbrain (RL: 0.50 and 0.55-fold by SD-C and SD-H, respectively), medulla (VII: 0.70 and 0.23; VNC: 0.48 and 0.93-fold by SD-C and SD-H, respectively), and fiber tracts (IIIn: not determined; ml: 0.48 and 0.98; ccg: 0.20 and 0.69; rust: 0.68 and 0.91; fx: 0.48 and 0.55-fold by SD-C and SD-H, respectively) compared with *ad libitum* sleep (Table 1). The expression ratios of cFos-TRAPed cells were also significantly changed in the thalamus (EPI: 0.67 and 3.37-fold by SD-C and SD-H, respectively) (Table 1). There was a significant interaction between the two variables in the striatum (CP), thalamus (EPI), hypothalamus (OV), and fiber tracts (ml and fx) (Table 1). Thus, both SD-H and SD-C increase cellular activity in the hypothalamus (LHA), midbrain (PBG and VTA), and medulla (MARN) but decrease it in the cortical subplate (BLAa), striatum (ACB, SH), thalamus (RT), hypothalamus (PVpo and VMPO), midbrain (RL), medulla (VII, VNC), and fiber tracts (IIIn, ccg, and rust). Consequently, in addition to the olfactory areas and medulla (Figure 3 and Supplementary Table S2), the cortical subplate, striatum, thalamus, hypothalamus, midbrain, and fiber tracts are the brain areas responsible for SD. No significant differences in the olfactory area were detected by the analysis of subdivided regions, indicating that the expression ratio of cFos-TRAPed cells was increased in a wide range of the olfactory areas but not in particular regions by SD.

**Table 1:** Ratio of cFos-TRAPed cells in subdivided brain regions and results of two-way ANOVA.

Brain region		Acronym	Ratio (# of cells in each class 6 region/# of cells in each class 1–5 region)				*p-*value by two-way ANOVA		
			*Ad lib* sleep		Sleep deprivation		Condition	Method	Condition × Method interaction
			Chamber	Hand	Chamber	Hand			
Olfactory areas	Other olfactory areas	Other OLF	3.820%	4.531%	3.680%	4.121%	0.0550	**0.0049**	0.2572
Cortical subplate	Claustrum	CLA	2.883%	7.357%	4.020%	4.724%	0.3987	**0.0309**	0.0761
	Basolateral amygdalar nucleus anterior part	BLAa	16.296%	15.120%	15.423%	11.745%	**0.0385**	**0.0256**	0.1481
Striatum	Caudoputamen	CP	27.470%	28.719%	31.707%	42.050%	**0.0007**	**0.0033**	**0.0079**
	Nucleus accumbens	ACB	8.389%	9.777%	5.885%	7.394%	**0.0012**	**0.0080**	0.8488
	Septohippocampal nucleus	SH	0.064%	0.063%	0.000%	0.013%	**0.0081**	0.6494	0.5938
	Other striatum	Other STR	7.498%	8.414%	10.562%	7.317%	0.1565	0.1081	**0.0211**
Pallidum	Globus pallidus external segment	GPe	9.399%	8.969%	11.484%	7.482%	0.6855	**0.0320**	0.0599
	Magnocellular nucleus	MA	2.856%	1.520%	3.176%	2.400%	0.0988	**0.0197**	0.3744
Thalamus	Reticular nucleus of the thalamus	RT	2.693%	1.693%	1.457%	1.219%	**0.0298**	0.0747	0.2142
	Epithalamus	EPI	1.576%	0.941%	1.051%	3.171%	**0.0204**	**0.0315**	**0.0038**
Hypothalamus	Anteroventral preoptic nucleus	AVP	0.195%	0.273%	0.781%	0.152%	0.0918	0.0592	**0.0283**
	Medial preoptic area	MPO	3.051%	3.803%	2.739%	4.916%	0.4270	**0.0321**	0.1916
	Vascular organ of the lamina terminalis	OV	0.016%	0.000%	0.100%	0.000%	**0.0126**	**0.0041**	**0.0126**
	Periventricular hypothalamic nucleus preoptic part	PVpo	1.421%	1.791%	0.630%	0.879%	**0.0069**	0.1355	0.7346
	Ventromedial preoptic nucleus	VMPO	0.687%	0.579%	0.037%	0.382%	**0.0073**	0.2315	0.0544
	Anterior hypothalamic nucleus	AHN	4.714%	5.904%	7.658%	4.289%	0.1290	**0.0353**	**0.0028**
	Lateral hypothalamic area	LHA	9.209%	9.078%	10.692%	10.790%	**0.0218**	0.9711	0.8070
	Preparasubthalamic nucleus	PST	0.460%	0.000%	0.151%	0.000%	0.0910	**0.0118**	0.0910
	Tuberal nucleus	TU	3.781%	3.291%	3.749%	2.130%	0.1289	**0.0279**	0.1453
Midbrain	Superior colliculus optic layer	SCop	5.812%	4.009%	3.863%	5.086%	0.4099	0.5732	**0.0332**
	Inferior colliculus dorsal nucleus	ICd	2.570%	4.477%	2.256%	5.311%	0.6170	**0.0067**	0.2978
	Parabigeminal nucleus	PBG	0.000%	0.000%	0.044%	0.012%	**0.0272**	0.1277	0.1277
	Ventral tegmental area	VTA	0.371%	0.434%	0.749%	0.772%	**0.0461**	0.7514	0.8823
	Superior colliculus motor related intermediate white layer	SCiw	8.014%	5.930%	8.213%	5.865%	0.9097	**0.0163**	0.8245
	Superior colliculus motor related intermediate gray layer	SCig	10.550%	7.829%	8.515%	7.814%	0.1142	**0.0282**	0.1181
	Medial terminal nucleus of the accessory optic tract	MT	0.039%	0.097%	0.157%	0.000%	0.6909	0.1292	**0.0144**
	Interpeduncular nucleus	IPN	0.424%	0.609%	0.249%	1.187%	0.1336	**0.0064**	**0.0247**
	Rostral linear nucleus raphe	RL	0.312%	0.182%	0.156%	0.101%	**0.0297**	0.0628	0.3605
Pons	Pontine gray	PG	35.926%	27.559%	23.416%	35.250%	0.5418	0.6569	**0.0492**
	Pontine reticular nucleus	PRNr	4.051%	3.631%	6.604%	2.657%	0.3591	**0.0458**	0.0819
	Other pons	Other pons	16.009%	24.533%	17.390%	25.570%	0.5952	**0.0164**	0.9386
Medulla	Facial motor nucleus	VII	6.44%	6.08%	4.48%	1.40%	**0.0410**	0.1988	0.2892
	Magnocellular reticular nucleus	MARN	1.61%	2.10%	2.27%	4.96%	**0.0255**	**0.0349**	0.0962
	Vestibular nuclei	VNC	12.17%	8.52%	5.88%	7.90%	**0.0358**	0.5045	0.0634
	Nucleus raphe magnus	RM	1.19%	0.74%	1.59%	0.10%	0.5941	**0.0091**	0.0645
Cerebellum	Lobule III	CENT3	15.618%	9.265%	20.029%	8.415%	0.5045	**0.0209**	0.3401
fiber tracts	oculomotor nerve	IIIn	0.174%	0.034%	0.000%	0.000%	**0.0164**	0.0548	0.0548
	brachium of the inferior colliculus	bic	0.528%	0.911%	1.898%	0.643%	0.1191	0.1930	**0.0424**
	medial lemniscus	ml	10.581%	5.553%	5.084%	5.453%	**0.0242**	**0.0424**	**0.0271**
	genu of corpus callosum	ccg	1.023%	0.525%	0.202%	0.364%	**0.0252**	0.2982	0.0792
	corticospinal tract	cst	0.000%	1.652%	0.000%	0.925%	0.4308	**0.0361**	0.4308
	internal capsule	int	4.575%	2.929%	4.141%	2.850%	0.6170	**0.0364**	0.7275
	rubrospinal tract	rust	1.913%	1.037%	1.299%	0.943%	**0.0407**	**0.0066**	0.0942
	columns of the fornix	fx	2.469%	1.217%	1.190%	0.663%	**0.0000**	**0.0000**	**0.0000**
	mammillary peduncle	mp	0.053%	0.211%	0.425%	0.065%	0.1547	0.1906	**0.0158**
ventricular systems	lateral recess	V4r	7.836%	9.559%	1.793%	15.837%	0.9674	**0.0436**	0.0852

The ratios of cFos-TRAPed cells are shown for regions with *p* < 0.05 by two-way ANOVA. All data are available in Supplementary Table S3. Bold values denote statistical significance at the *p*<0.05 level.

Compared with group C, group H showed significantly higher expression ratios of cFos-TRAPed cells in subdivided regions including the olfactory areas (other olfactory areas), cortical subplate (CLA), striatum (CP and ACB), thalamus (EPI), hypothalamus (MPO), midbrain (ICd and IPN), pons (other pons), medulla (MARN), fiber tracts (cst), and ventricular systems (V4r), but were significantly lower in the cortical subplate (BLAa), pallidum (GPe and MA), hypothalamus (OV, AHN, PST, and TU), midbrain (SCiw and SCig), pons (PRNr), medulla (RM), cerebellum (CENT3), and fiber tracts (ml, int, rust, and fx). In addition, the six subdivided regions showed significant interactions between the two variables [striatum (CP), thalamus (EPI), hypothalamus (OV and AHN), midbrain (IPN), and fiber tracts (ml)]. Thus, the cellular activity in groups H and C was differentially affected in the olfactory areas (other olfactory areas), cortical subplate (CLA and BLAa), striatum (ACB), pallidum (GPe and MA), hypothalamus (MPO, PST, and TU), midbrain (ICd, SCiw, and SCig), pons (PRNr and other pons), medulla (MARN and RM), cerebellum (CENT3), fiber tracts (cst, int, rust, and fx), and ventricular systems (V4r). Detailed comparisons between groups H and C are shown in Table 1. Significant differences were also observed in the interactions between the two variables, but not between each variable, in the striatum (other striatum), hypothalamus (AVP), midbrain (Scop, MT), pons (PG), and fiber tracts (bic and mp). However, none of these subdivided regions were obtained by the above analyses.

### 3.4. Sleep deprivation affects connectivity among brain regions

**Figure 4:**
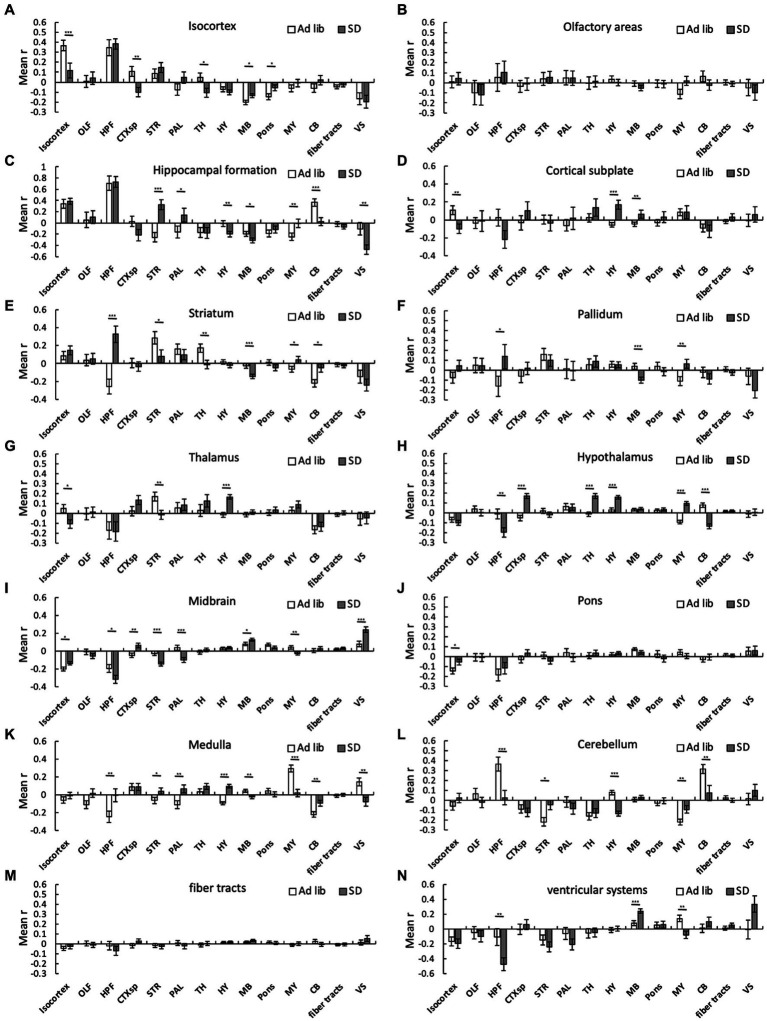
Connectivity changes among brain regions due to SD. Connectivity among 294 regions was estimated for *ad libitum* sleep animals and SD animals regardless of the method, and mean interregional correlation coefficients (mean r) were obtained for the 14 brain regions. The bar graph shows the mean r between one region and another within that region: **(A)**, isocortex; **(B)**, olfactory areas; **(C)**, hippocampal formation; **(D)**, cortical subplate; **(E)**, striatum; **(F)**, pallidum; **(G)**, thalamus; **(H)**, hypothalamus; **(I)**, midbrain; **(J)**, pons; **(K)**, medulla; **(L)**, cerebellum; **(M)**, fiber tracts; and **(N)** ventricular systems. Error bars indicate SEM. *p < 0.05; **p < 0.01; and ***p < 0.001 for the difference between *ad libitum* sleep mice and SD mice by Kolmogorov–Smirnov test.

We then analyzed the changes in connectivity among the brain regions due to SD. SD alters connectivity in several cross-brain regions. For instance, the mean r in the sum_SD group was significantly higher than that in the sum_ad libitum sleep group in the following cross-brain regions: isocortex-midbrain, isocortex-pons, hippocampal formation-striatum, hippocampal formation-pallidum, hippocampal formation-medulla, cortical subplate-hypothalamus, cortical subplate-midbrain, striatum-medulla, striatum-cerebellum, pallidum-medulla, thalamus-hypothalamus, hypothalamus-hypothalamus, hypothalamus-medulla, midbrain-midbrain, medulla-cerebellum, and medulla-ventricular systems (Figure 4). In contrast, the mean r in the sum_SD group was significantly lower than that in the sum_ad libitum sleep group in the following cross-brain regions: isocortex-isocortex, isocortex-cortical subplate, isocortex-thalamus, hippocampal formation-hypothalamus, hippocampal formation-midbrain, hippocampal formation-cerebellum, hippocampal formation-ventricular systems, striatum-striatum, striatum-thalamus, striatum-midbrain, pallidum-midbrain, hypothalamus-cerebellum midbrain-medulla, medulla-medulla, medulla-ventricular systems, and cerebellum-cerebellum (Figure 4).

Connectivity in several cross-brain regions was also differentially altered in groups C and H. The mean r in sum_hand was significantly higher than that in sum_chamber in the following cross-brain regions: isocortex-hypothalamus, olfactory areas-medulla, cortical subplate-hypothalamus, cortical subplate-pons, cortical subplate-medulla, striatum-striatum, striatum-pallidum, striatum-hypothalamus, thalamus-hypothalamus, thalamus-fiber tracts, hypothalamus-hypothalamus, hypothalamus-medulla, hypothalamus-fiber tracts, midbrain-ventricular systems, and medulla-cerebellum (Supplementary Figure S1). Conversely, the mean r in sum_hand was significantly lower than that in sum_chamber in the following cross-brain regions: isocortex-isocortex, isocortex-cortical subplate, olfactory areas-olfactory areas, olfactory areas-hippocampal formation, olfactory areas-cortical subplate, olfactory areas-hypothalamus, hippocampal formation-medulla, hippocampal formation-cerebellum, cortical subplate-cortical subplate, striatum-midbrain, striatum-cerebellum, striatum-ventricular systems, pallidum-midbrain, pallidum-cerebellum, hypothalamus-midbrain, hypothalamus-cerebellum, and medulla-medulla (Supplementary Figure S1).

## 4. Discussion

Consistent with previous studies, we observed significant increases in cFos-TRAPed cells due to SD in the caudoputamen (striatum) (Cirelli et al., 1995; Semba et al., 2001), epithalamus (thalamus) (Cirelli et al., 1995), lateral hypothalamic area (hypothalamus) (Modirrousta et al., 2005), parabigeminal nucleus (midbrain) (Pompeiano et al., 1992), and ventral tegmental area (midbrain) (Maloney et al., 2002). Recent studies have revealed that all of these regions are involved in the sleep/wake regulatory circuit. For instance, it has been reported that the level of dorsal striatal (caudoputamen) dopamine correlates with the sleep–wake cycle and responds to modafinil (Dong et al., 2019). The epithalamus contains a pineal gland that secretes melatonin. Orexin neurons are mainly located in the lateral hypothalamic area and play a role in arousal regulation (Saper et al., 2005; Pizza et al., 2022). Furthermore, GABAergic neurons in the ventral tegmental area regulate sleep–wake behavior (Takata et al., 2018; Yu et al., 2019). Our study also showed that the number of cFos-TRAPed cells decreased in several brain regions that modulate sleep–wake behavior due to SD. These regions are the amygdala (cortical subplate) (Hasegawa et al., 2022), nucleus accumbens (striatum) (Oishi et al., 2017; Luo et al., 2018), septohippocampal nucleus (striatum) (Wu et al., 2004), reticular nucleus of the thalamus (thalamus) (Vantomme et al., 2019; Liu et al., 2021), periventricular nucleus (hypothalamus) (Sakai, 2011), ventromedial preoptic nucleus (hypothalamus) (Ikeda et al., 2001), rostral linear nucleus raphe (midbrain) (Jolas and Aghajanian, 1997), and vestibular nuclei (medulla) (Bizzi et al., 1964; Besnard et al., 2018). The evidence that SD by both the methods induces activation of the olfactory areas on common with the two methods suggests that this activation is due to SD and not the experimenter’s odor. Additionally, we observed a significant decrease and increase in cFos-TRAPed cells in the facial motor nucleus and magnocellular reticular nucleus (both in the medulla), respectively, due to SD. These two medullary regions may be involved in the regulation of sleep/wake patterns, although their role in SD has not yet been reported.

Most of the previous studies examining cFos expression in SD reported only brain regions where cFos expression is increased (Merchant-Nancy et al., 1992; O’Hara et al., 1993; Cirelli et al., 1995; Semba et al., 2001; Terao et al., 2003; Jha et al., 2017; Montes-Rodríguez et al., 2019; Cai et al., 2022). For example, Cai et al. applied a comprehensive mapping of cumulatively activated brain regions in chronic REM SD mice and found that SD increased the proportion of cFos-expressing cells in the lateral habenula of the epithalamus (Cai et al., 2022), which is consistent with the findings of our study. However, none of the other regions were commonly detected in these two studies. This could be attributed to the fact that the investigators evaluated only brain regions that showed cumulative expression levels of cFos during the entire chronic REM SD process. Given that neurons in some brain regions are activated during sleep (Saper et al., 2005; Rothhaas and Chung, 2021) or the period when switching between NREM and REM sleep (Weber et al., 2015; Corsi-Cabrera et al., 2016; Oishi et al., 2017; Latifi et al., 2018; Ma et al., 2019; Li et al., 2021; Song and Zhu, 2021; Dong et al., 2022; Hasegawa et al., 2022), evaluating both activated and deactivated cellular responses to SD would be necessary to understand the detailed mechanisms by which acute/chronic SD and sleep disruption affect brain function.

In this study, we analyzed the correlation of cFos expression among brain regions and estimated connectivity of cellular activity among 14 brain regions using mean r (Wheeler et al., 2013; Tanimizu et al., 2018). Significant changes in mean r due to SD were observed in several brain regions (Figure 4), suggesting that mean r may be useful for evaluating connectivity in response to certain physiological conditions. However, it should be noted that the mean r of each region reflects the fact that positive or negative correlations dominate rather than the strength of the correlation due to the existence of both positive and negative correlations in each brain region. In contrast, the means of the absolute values of r, positive values of r, and negative values of r reflect the strength of the correlation (Supplementary Figures S2A–N, S3A–N). Nevertheless, to the best of our knowledge, this is the first study to show brain network changes due to SD in rodents. Comparison of these results with other approach such as fMRI in future studies is important. Several fMRI studies have so far shown that SD causes brain network changes in humans (Gujar et al., 2010; De Havas et al., 2012; Bosch et al., 2013; Shao et al., 2013; Dai et al., 2015; Zhu et al., 2016; Tagliazucchi and van Someren, 2017; Zhou et al., 2017; Chen et al., 2018; Wirsich et al., 2018; Ye et al., 2018). For instance, when focusing on default mode networks, which refer to the brain regions that are more active during the resting state, SD promotes not only reduced connectivity, but also compensatory increase in connectivity (Gujar et al., 2010; De Havas et al., 2012; Chen et al., 2018; Wirsich et al., 2018). The default mode network is known to be associated with brain functions such as cognition, emotion, and decision making (Satpute and Lindquist, 2019; Smallwood et al., 2021). Neuropsychiatric disorders can also cause abnormalities in the default mode network (Whitfield-Gabrieli and Ford, 2012). The default mode network regions are mainly within the isocortex in rodents (Whitesell et al., 2021). Consistent with fMRI studies in humans, we found significant changes in connectivity within the isocortex due to SD in mice. Therefore, elucidating connectivity, especially within the isocortex, using TRAP2 mice may support the evidence from fMRI studies.

Analyses from multiple perspectives using the same individual tissue helped us fully evaluate brain function. Tissue clearing is often combined with labeling techniques to directly count activated neurons (Liang and Luo, 2021). Although tissue clearing is a fascinating tool for whole-brain evaluation, it requires the whole brain of each animal. One advantage of our method is that multiple analyses can be conducted using serial sections from the same individual. Analyses from multiple perspectives are often required to fully understand the individual physiology when mice are exposed to interventions. This advantage is also applicable when considering the limitation of mouse resources (e.g., aged mice, genetically engineered mouse model by stereotactic injection) and the 3Rs of animal research, which are superior from the point of view of laboratory animal welfare as they reduce the number of animals used.

Our study has some methodological limitations. First, registration of sections to the atlas is a time-consuming procedure and needs attention to detail, such as the angle of the sections, and structural changes associated with section preparation. Lauridsen et al. pointed out that uneven brain ventricular size, resulting from brain ventricular dilatation during perfusion, makes accurate atlas registration difficult. In this study, misplacement of cells in the atlas was minimized by manual adjustment of cell coordinates, as the SHARCQ allows manual adjustment of cell positions after registration (Lauridsen et al., 2022). Second, caution should be taken when comparing brain regions where the size is relatively small or where a small number of cFos is expressed. We observed SD-dependent changes in the expression ratios and connectivity of the fiber tract and ventricular systems. A few cFos-TRAPed cells (4.3%) were observed in the fiber tract, where a few cell bodies were present. However, these significant differences may be misdirected because of the relatively small size of the fiber tract and the irregular shape of the ventricular systems that make it difficult to accurately register to the atlas. Third, some regions affected by SD may not have been detected due to the small sample size (low statistical power). The overall statistical power of detected differences was between 0.4 and 0.8. While increasing the sample size may permit the detection of additional regions that are affected by SD, we believe that our study reasonably detected SD-related regions that have been consistently published in other papers. Additionally, regions that showing statistical differences, despite the small sample size, are undoubtedly affected by SD. Fourth, although most of the TRAPed cells were presumably captured when 4-OHT was injected, there is a possibility that the TRAPed cells also included those captured during recovery sleep. In this study, we intentionally selected the 5-h time point during SD for 4-OHT injection to completely eliminate any initial reactions induced by the experimental procedure that could affect neuronal activity unrelated to sleep loss (e.g., anxiety).

## 5. Conclusion

Comprehensive analysis of the whole brain using the TRAP technique with semi-automated registration SHARCQ. revealed that the number of cFos-TRAPed cells was significantly increased or decreased in several brain regions that have been reported to be involved in sleep/wake circuits. Such a comprehensive analysis of the whole brain is also useful for evaluating connectivity among brain regions. Our study provides novel insights into SD-induced brain activity changes and is potentially useful for understanding the underlying mechanisms of the pathophysiology related to sleep loss.

## Data availability statement

The original contributions presented in the study are included in the article/Supplementary material, further inquiries can be directed to the corresponding author.

## Ethics statement

The animal study was approved by the Animal Care and Use Committee at National Center for Geriatrics and Gerontology. The study was conducted in accordance with the local legislation and institutional requirements.

## Author contributions

TU and AS designed the research, wrote the manuscript, and analyzed the data. TU, MG, KK, MK, SM, ST, HT, and AS performed the research. All authors contributed to the article and approved the submitted version.
