# Supplementary material for: Evaluation of cellular activity in response to sleep deprivation by a comprehensive analysis of the whole mouse brain
Source: Front Neurosci. 2023 Oct 19;17:1252689. doi: 10.3389/fnins.2023.1252689 (PMC10620513; doi:10.3389/fnins.2023.1252689)
Supplement: Supplementary file 1 [file Data_Sheet_1.docx]

Supplementary Material

Evaluation of cellular activity in response to sleep deprivation by a comprehensive analysis of the whole mouse brain

Takuya Urushihata, Mio Goto, Keiko Kabetani, Mai Kiyozuka, Shiho Maruyama, Shogo Tsuji, Hirobumi Tada, and Akiko Satoh^*^

*** Correspondence:** Akiko Satoh, Ph.D: akiko.satoh.b7@tohoku.ac.jp


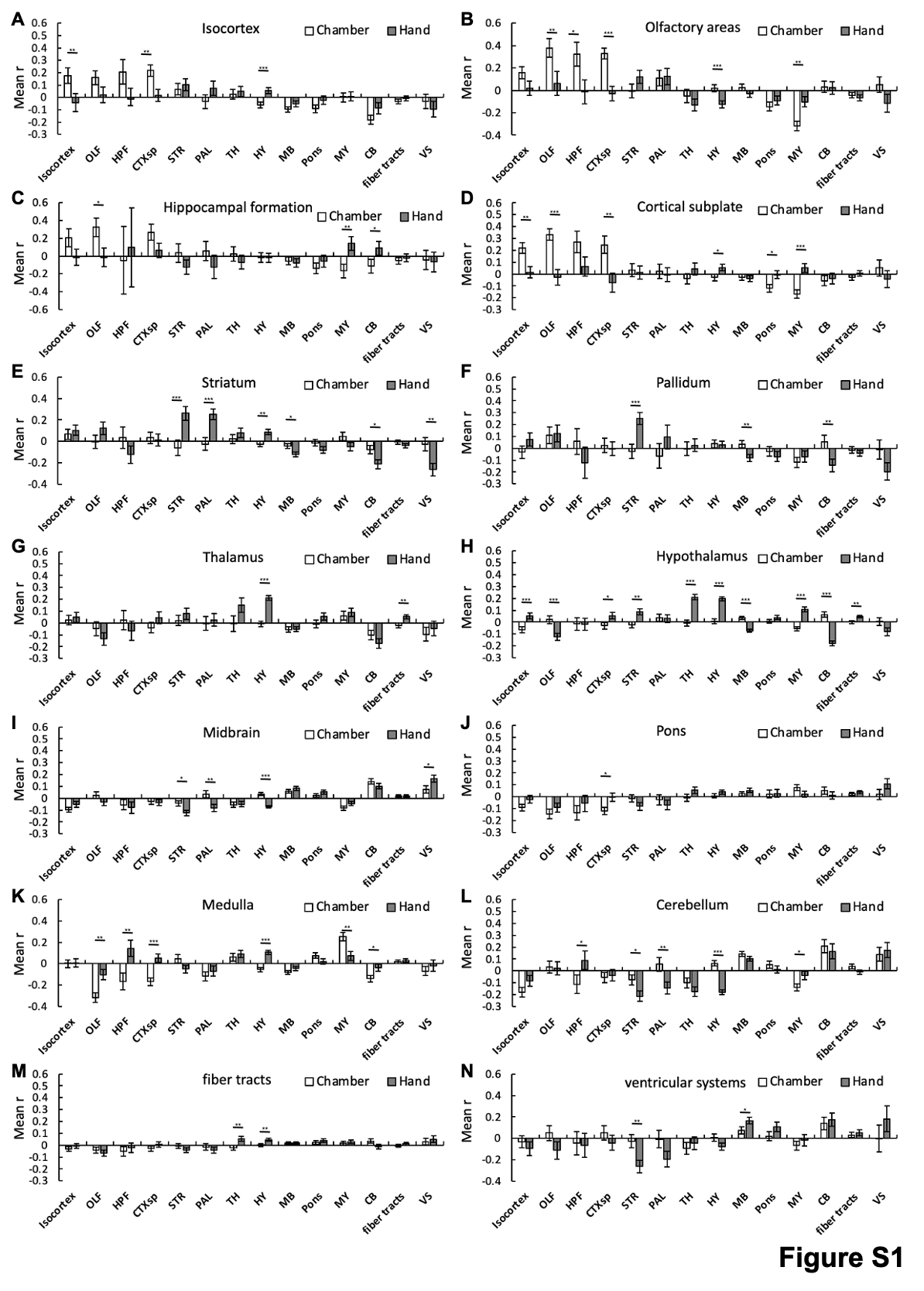


**Supplementary Figure S1** Connectivity changes among brain regions due to conditions.

Connectivity among 294 regions was estimated for animals in gentle hand and chamber conditions regardless of SD, and mean interregional correlation coefficients (mean r) were obtained for the 14 brain regions. The bar graph shows the mean r between one region and another region and within that region: **(A)** isocortex; **(B)** olfactory areas; **(C)** hippocampal formation; **(D)** cortical subplate; **(E)** striatum; **(F)** pallidum; **(G)** thalamus; **(H)** hypothalamus; **(I)** midbrain; **(J)** pons; **(K)** medulla; **(L)** cerebellum; **(M)** fiber tracts; and **(N)** ventricular systems. Error bars indicate SEM. *, *p* < 0.05; **, *p* < 0.01; and ***, *p* < 0.001 for the difference between gentle hand and chamber conditions by Kolmogorov-Smirnov test.


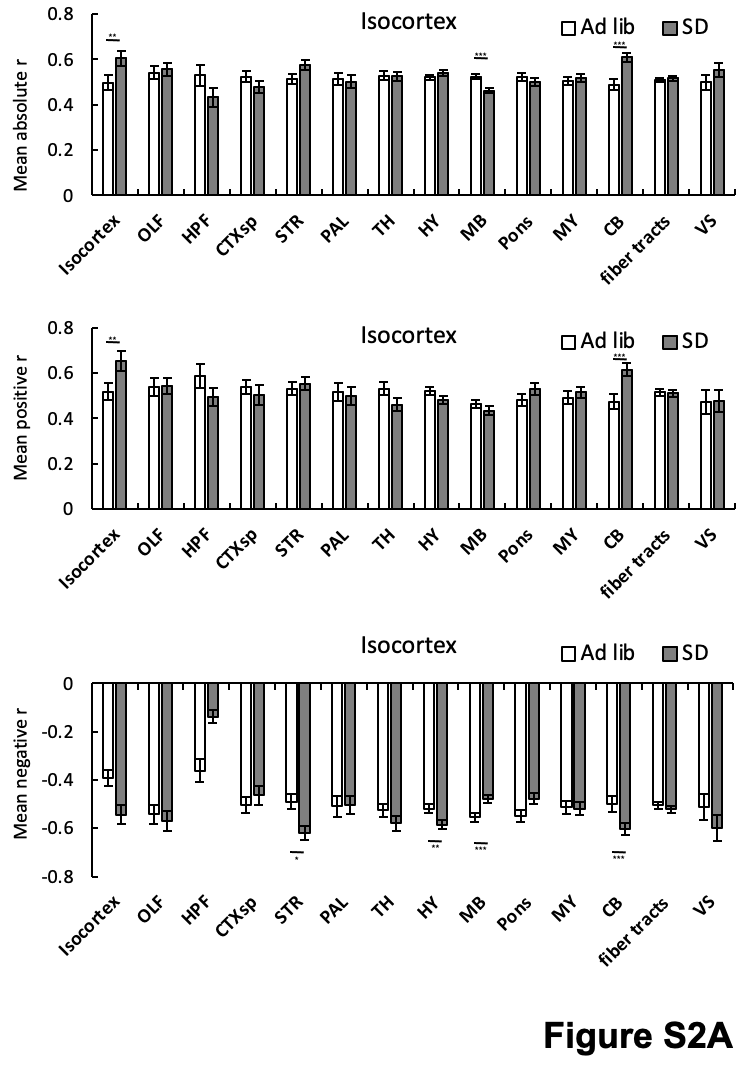


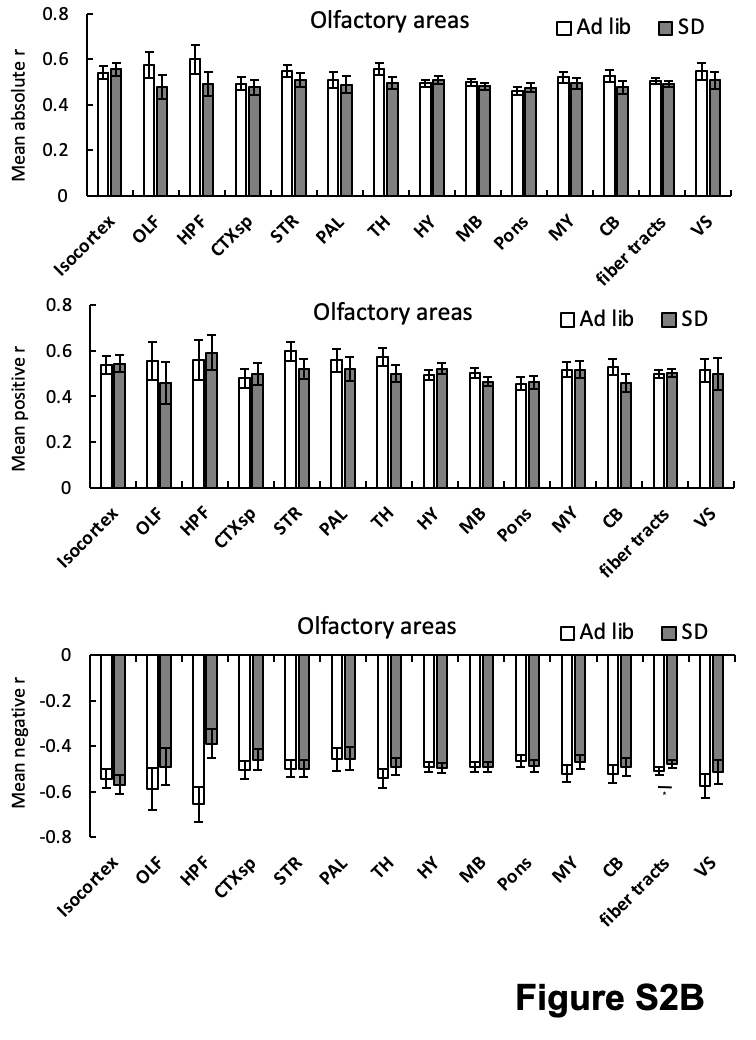


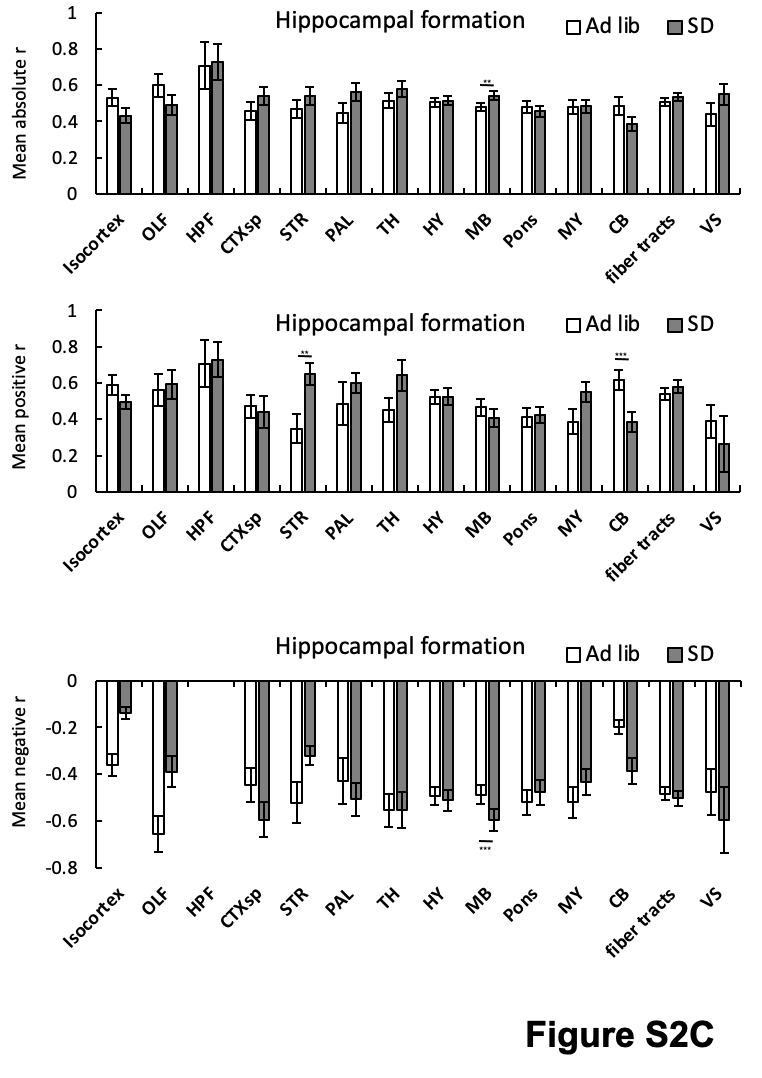


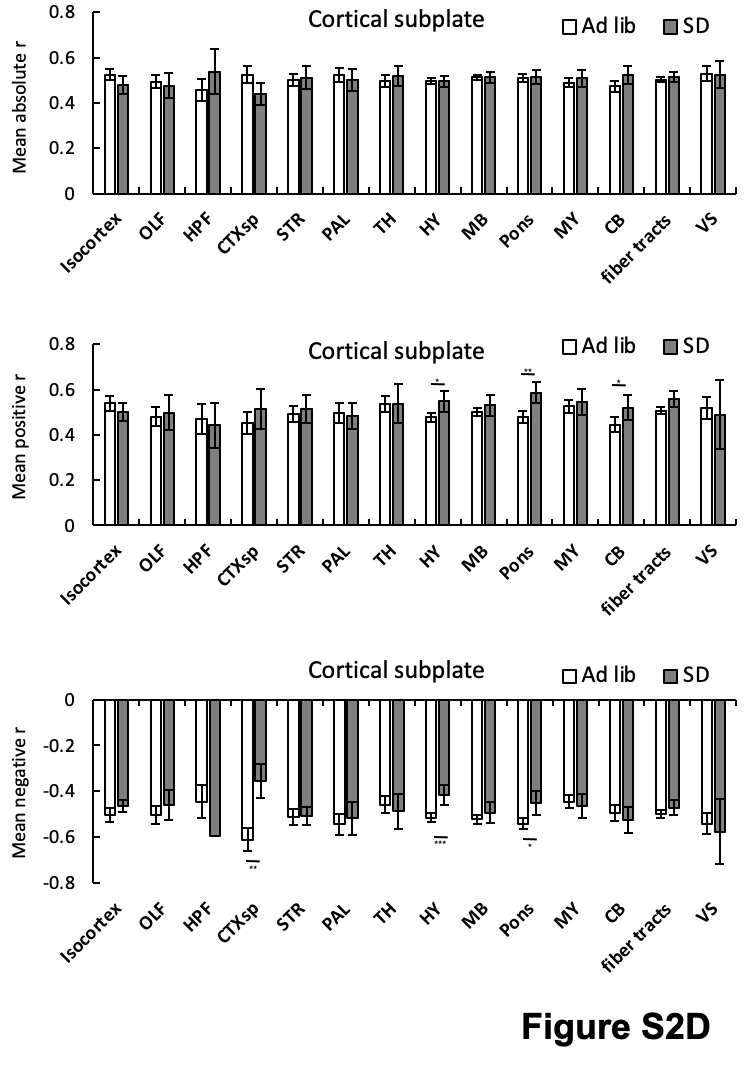


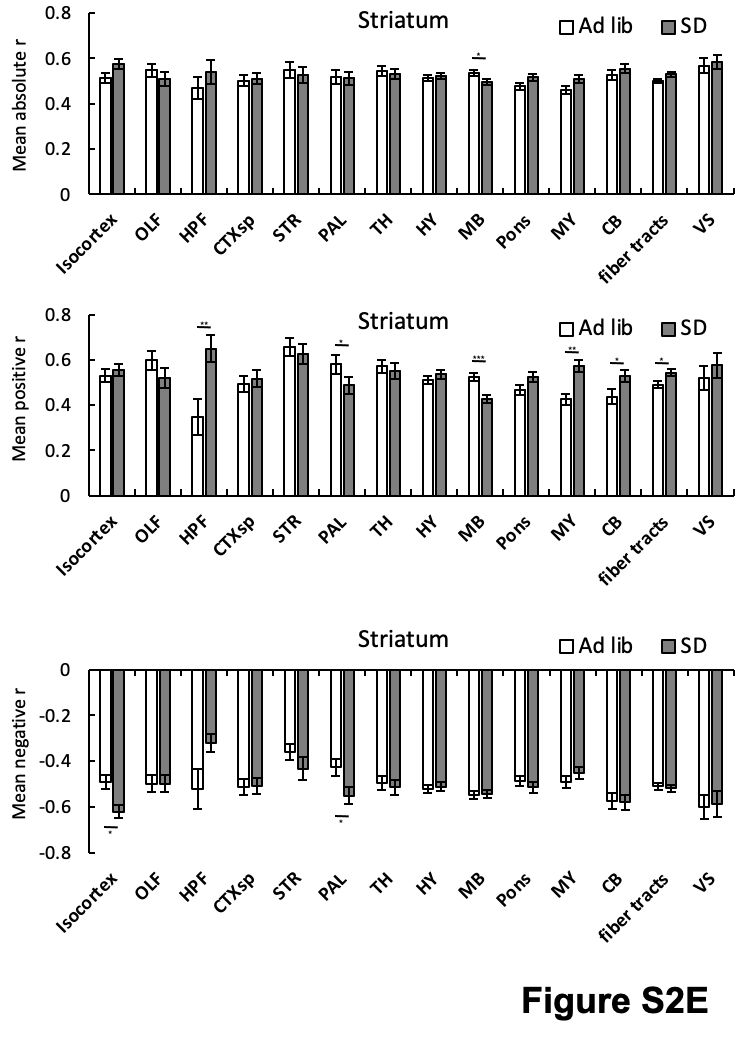


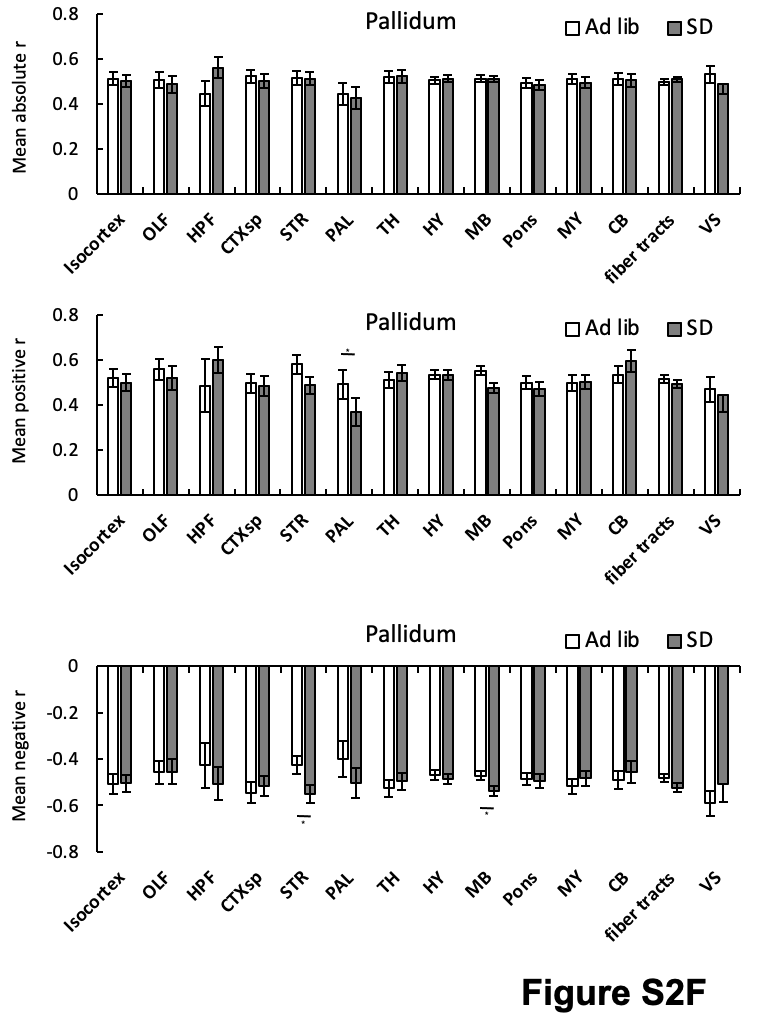


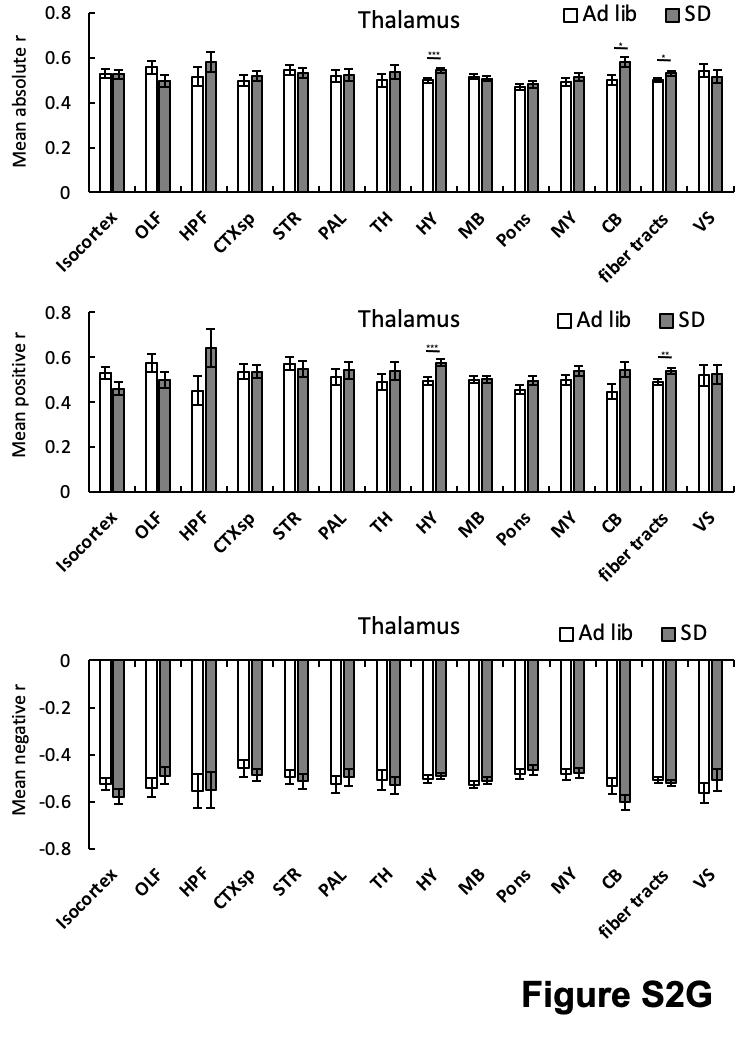


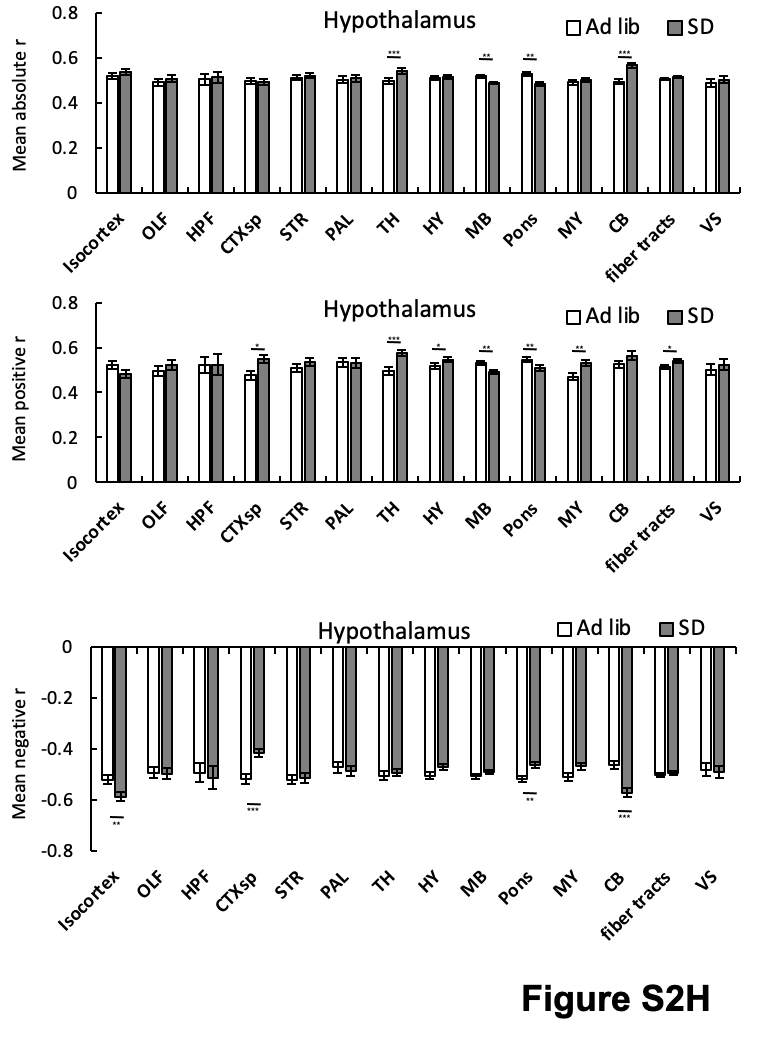


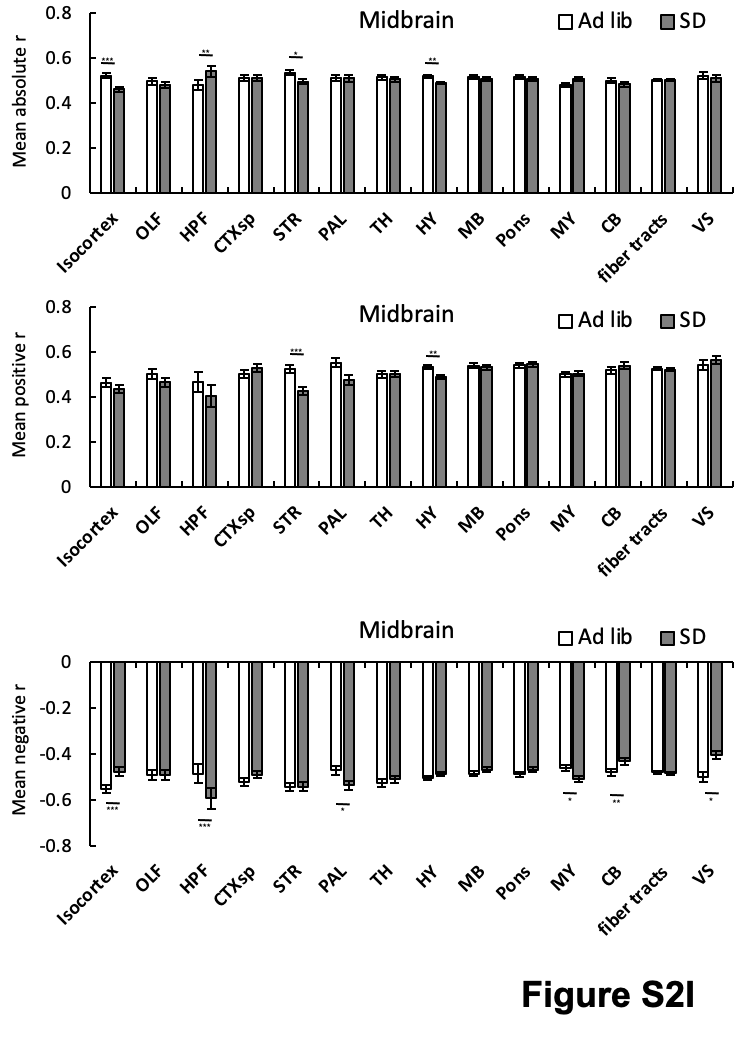


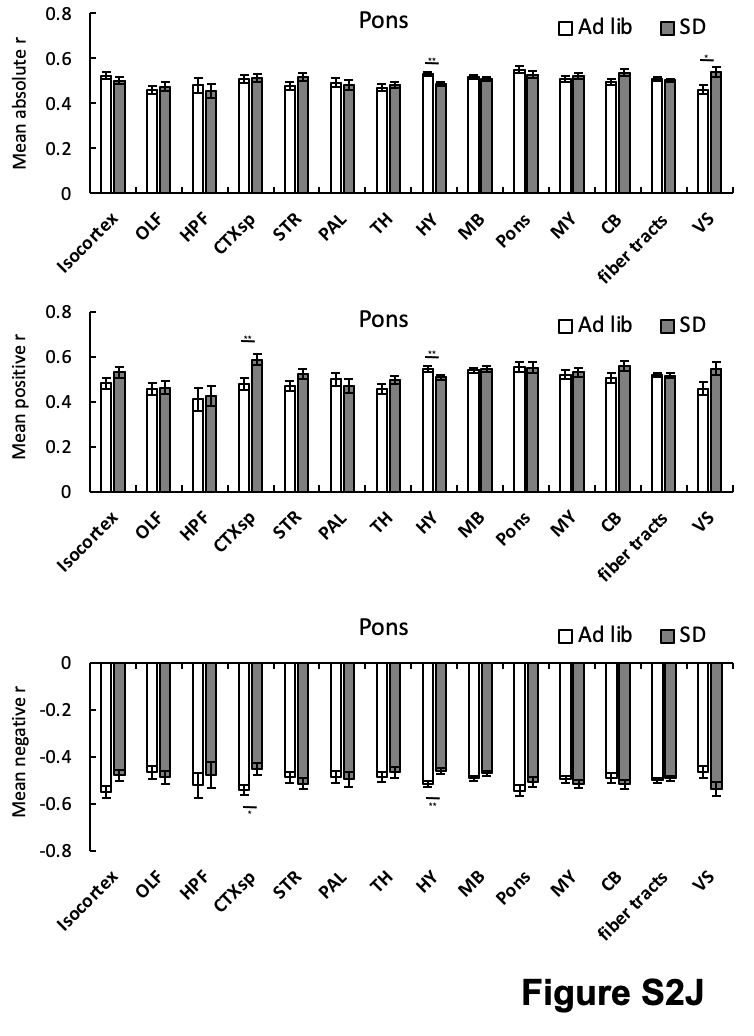


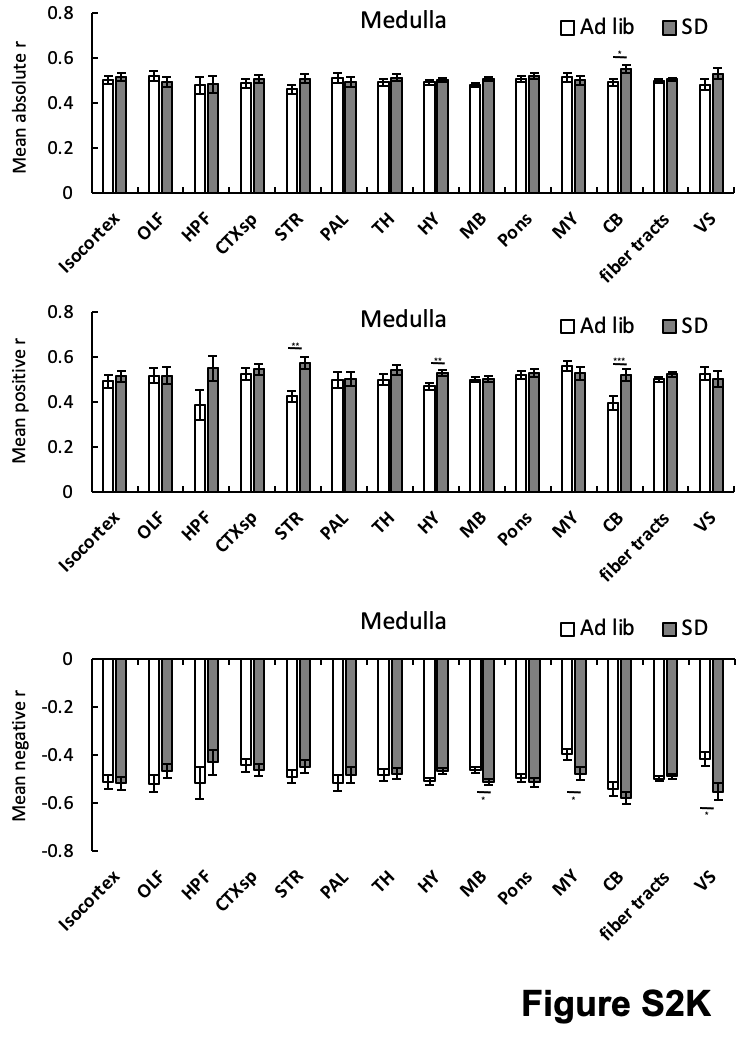


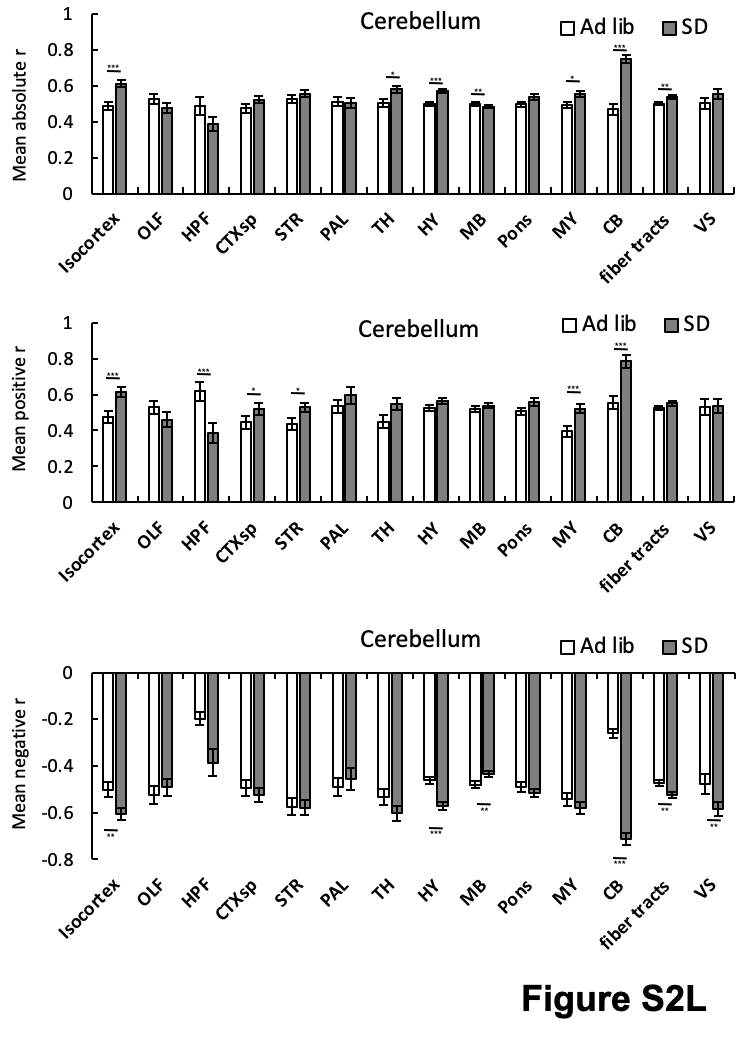


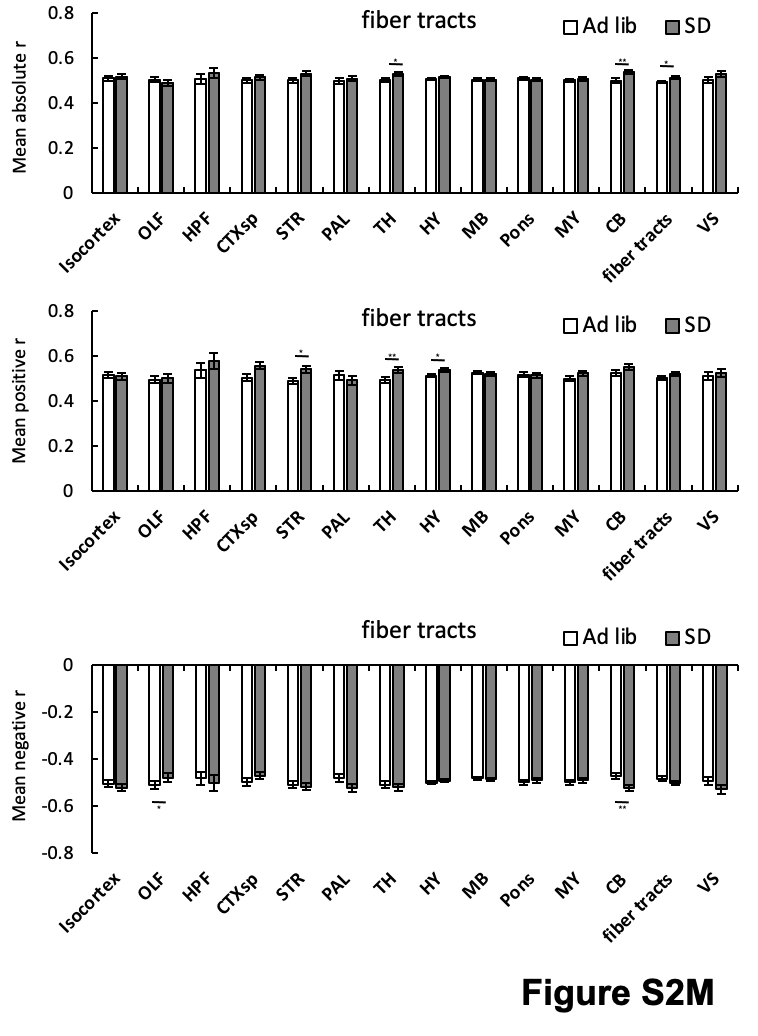


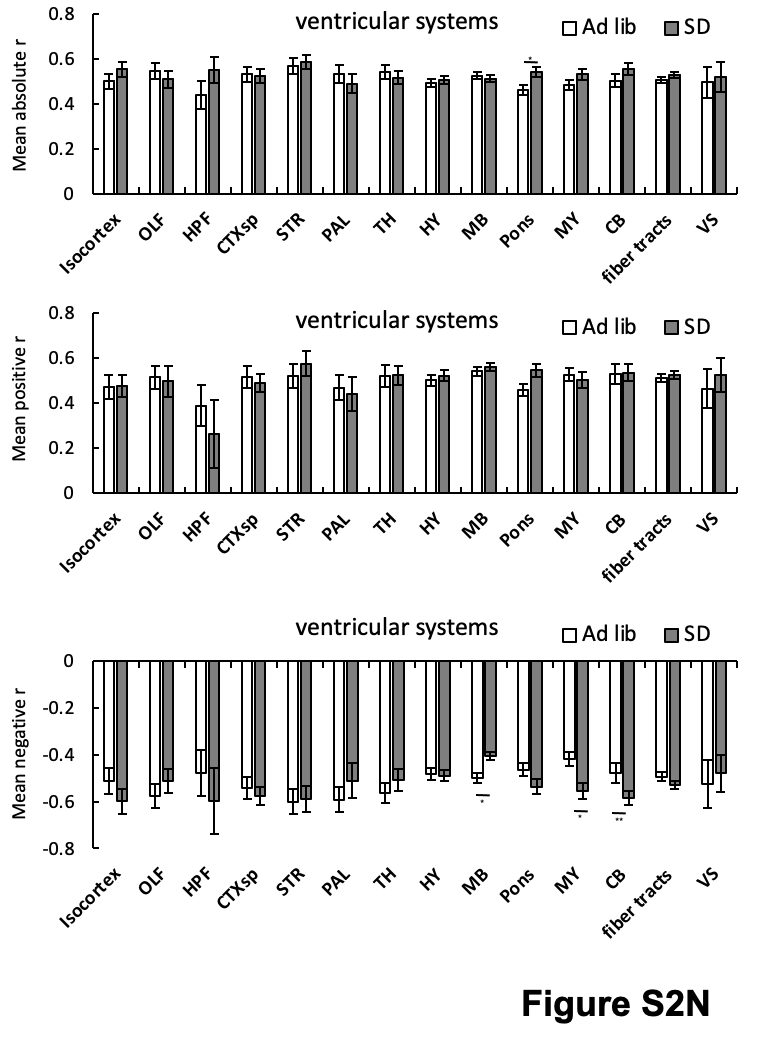


**Supplementary Figure S2** Conditional mean r changes for connectivity among brain regions due to SD.

Three conditional mean r for connectivity among brain regions, mean absolute r (top), mean positive r (middle), and mean negative r (bottom) are shown for each 14 regions, **(A)** isocortex; **(B)** olfactory areas; **(C)** hippocampal formation; **(D)** cortical subplate; **(E)** striatum; **(F)** pallidum; **(G)** thalamus; **(H)** hypothalamus; **(I)** midbrain; **(J)** pons; **(K)** medulla; **(L)** cerebellum; **(M)** fiber tracts; and **(N)** ventricular systems. Error bars indicate SEM. *, *p* < 0.05; **, *p* < 0.01; and ***, *p* < 0.001 for the difference between ad libitum sleep mice and SD mice by Kolmogorov-Smirnov test.


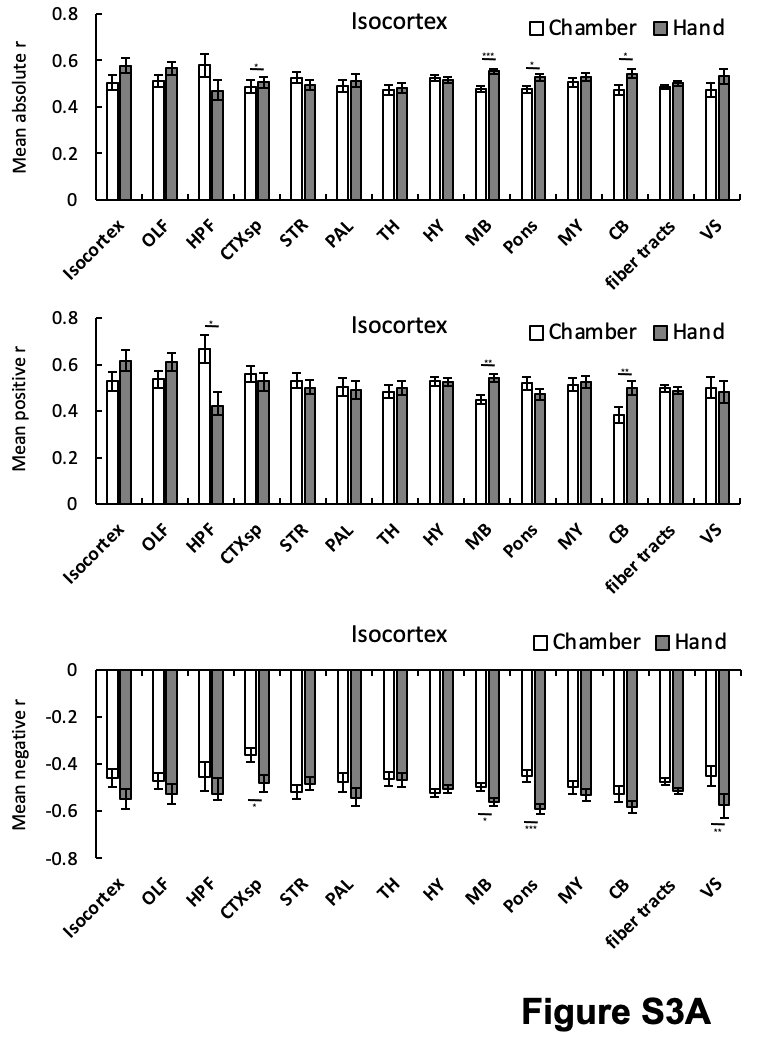


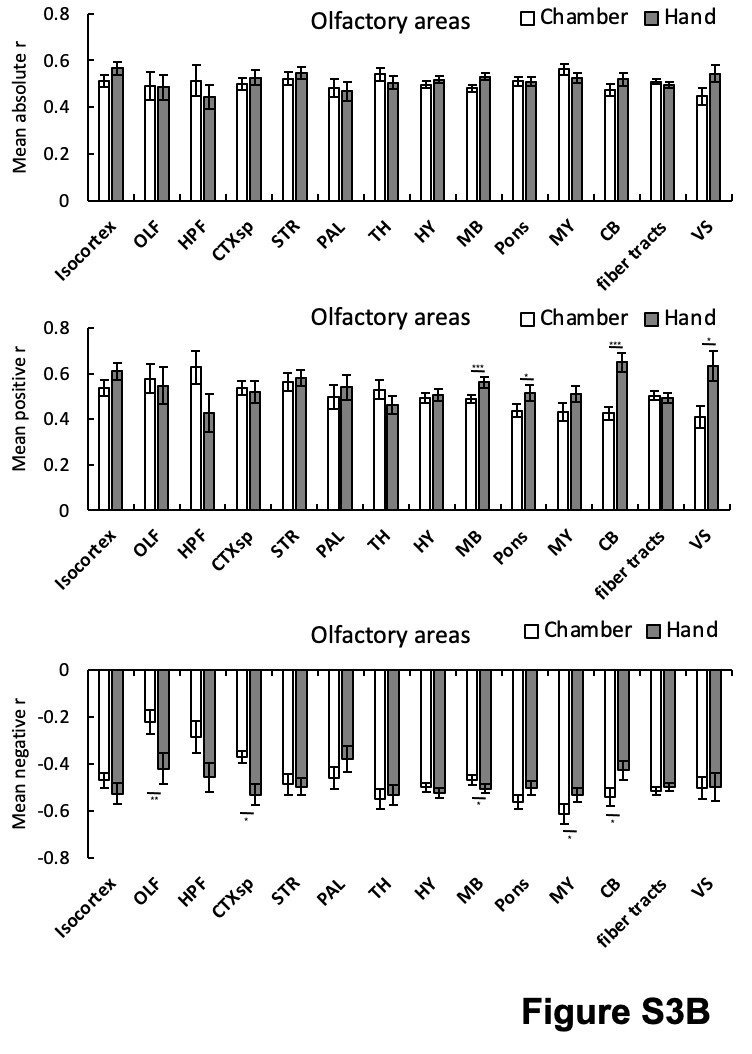


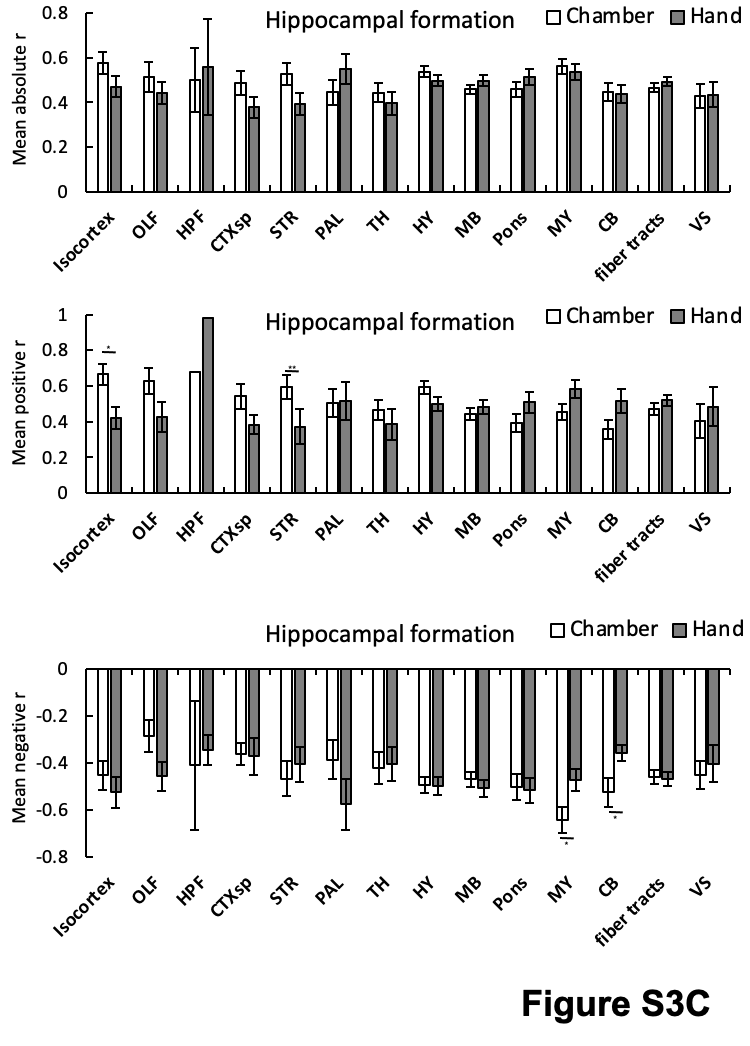


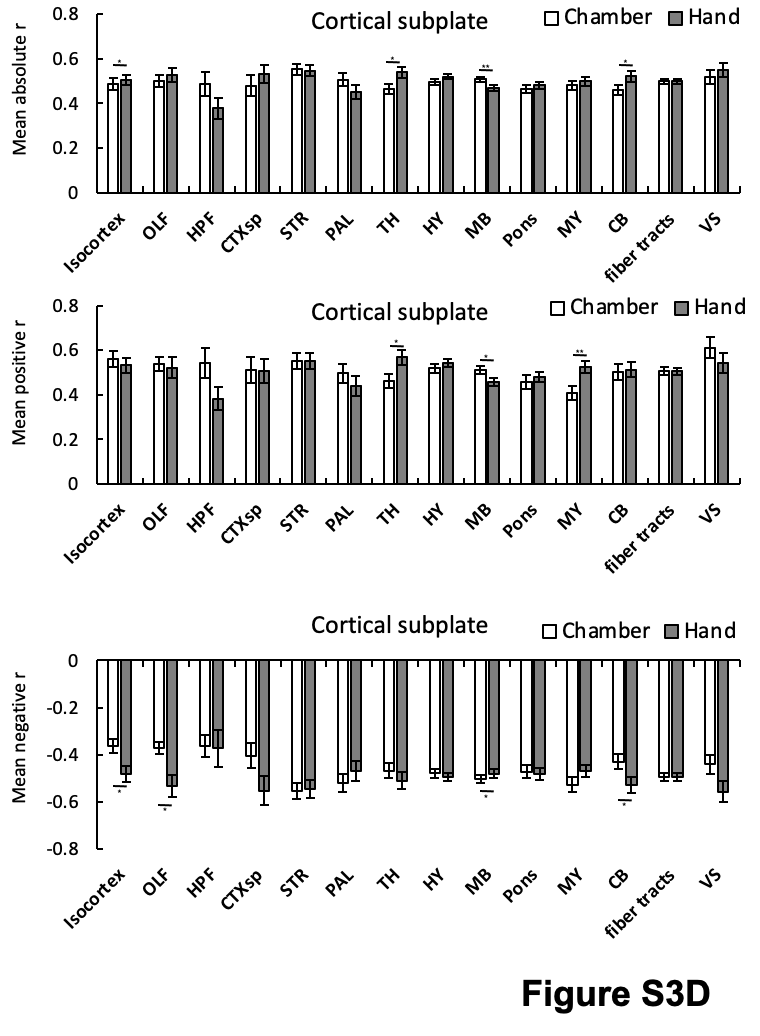


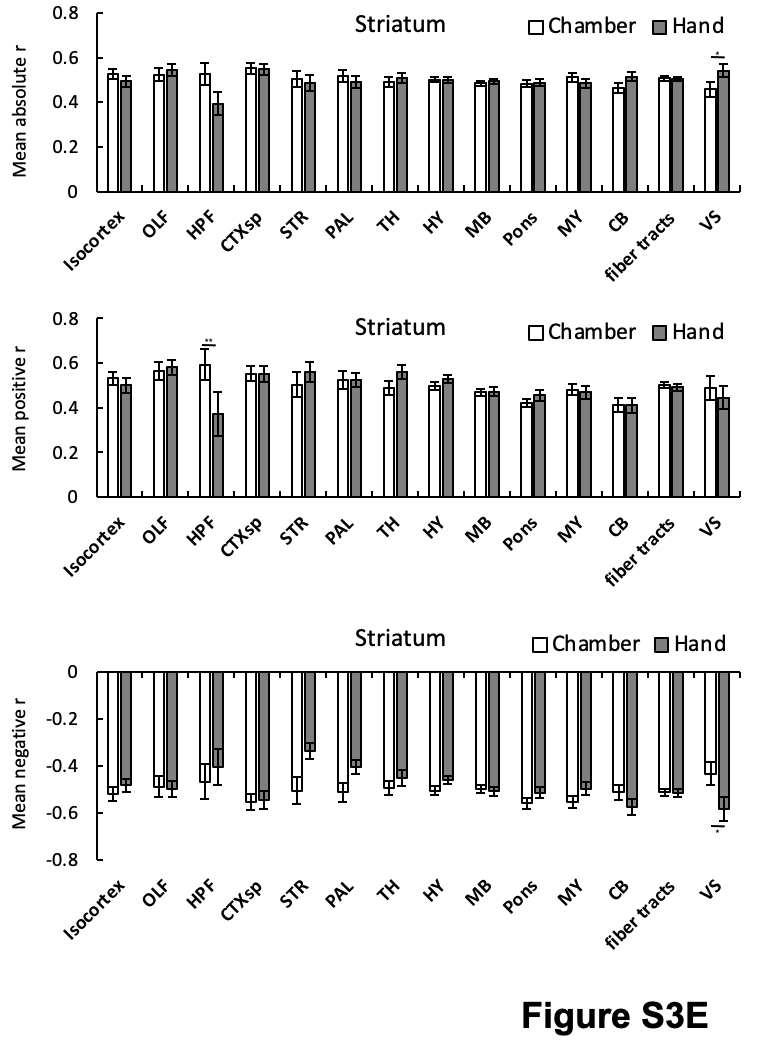


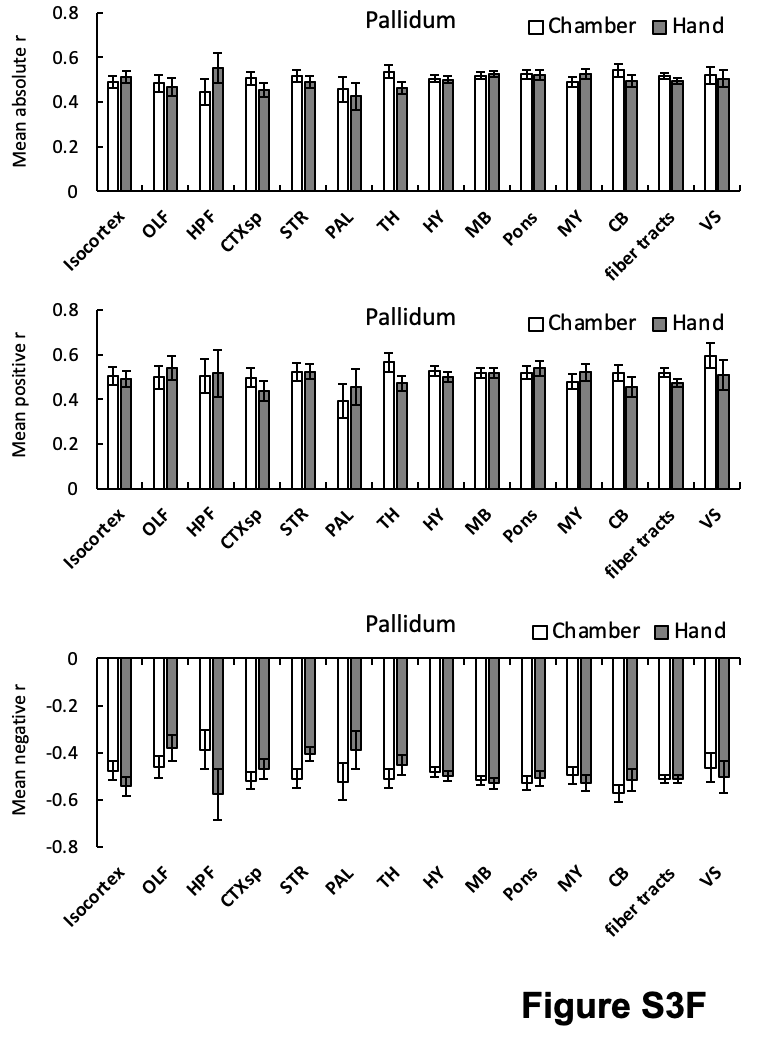


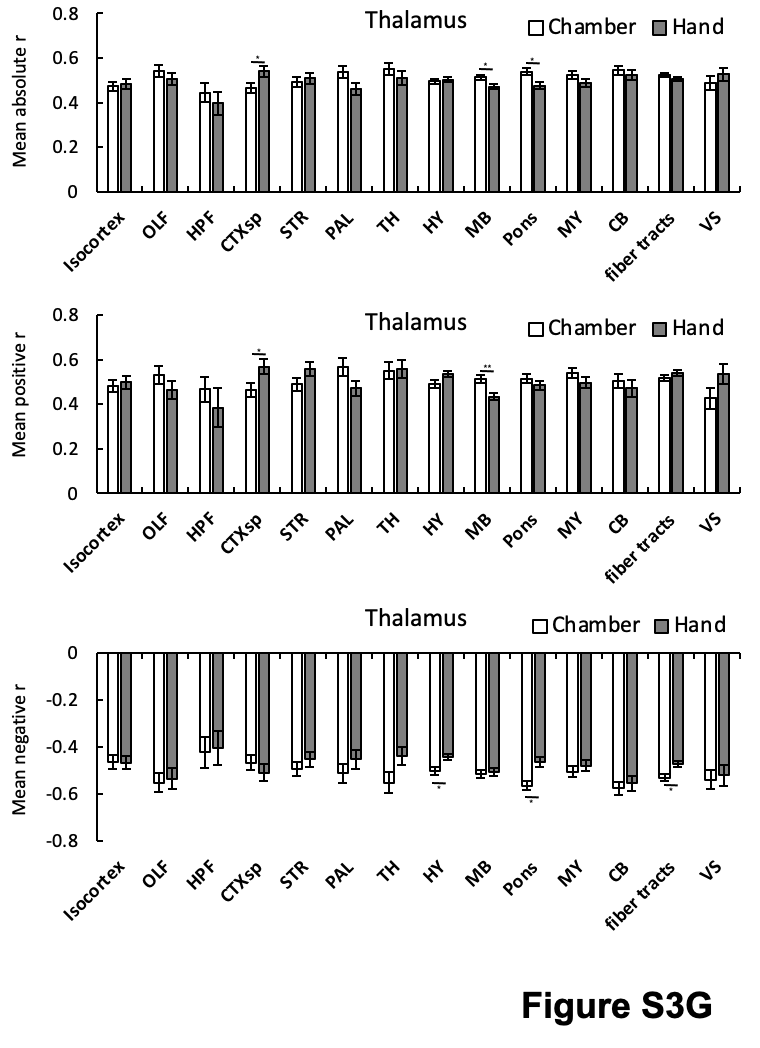


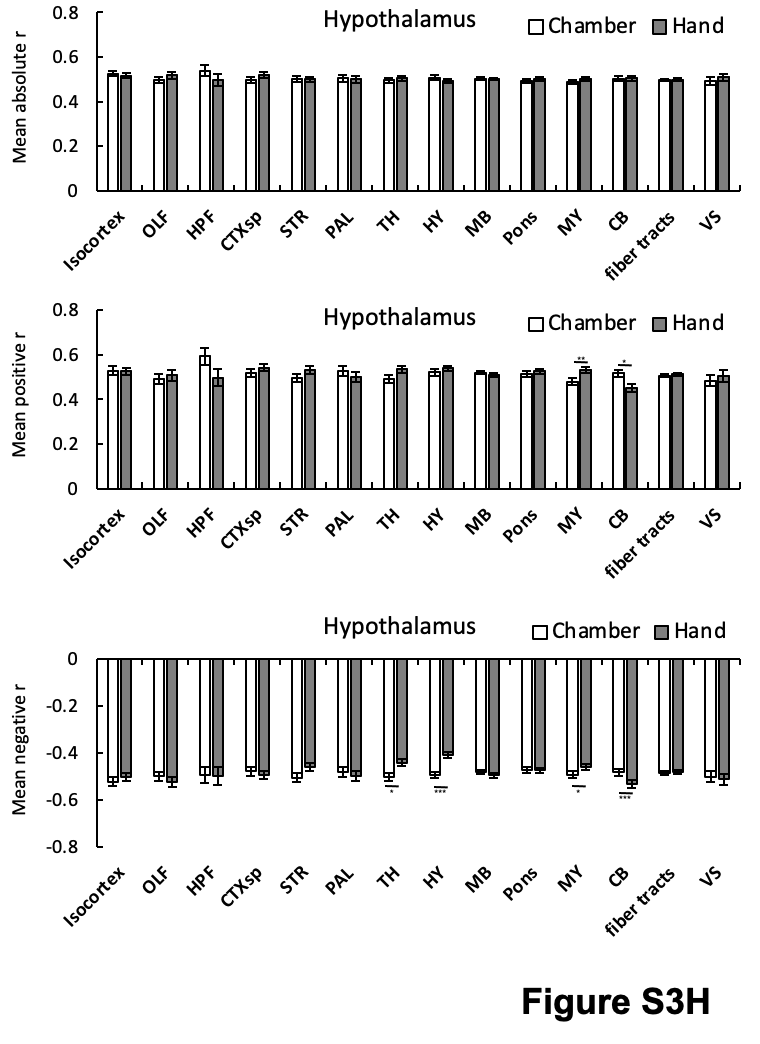


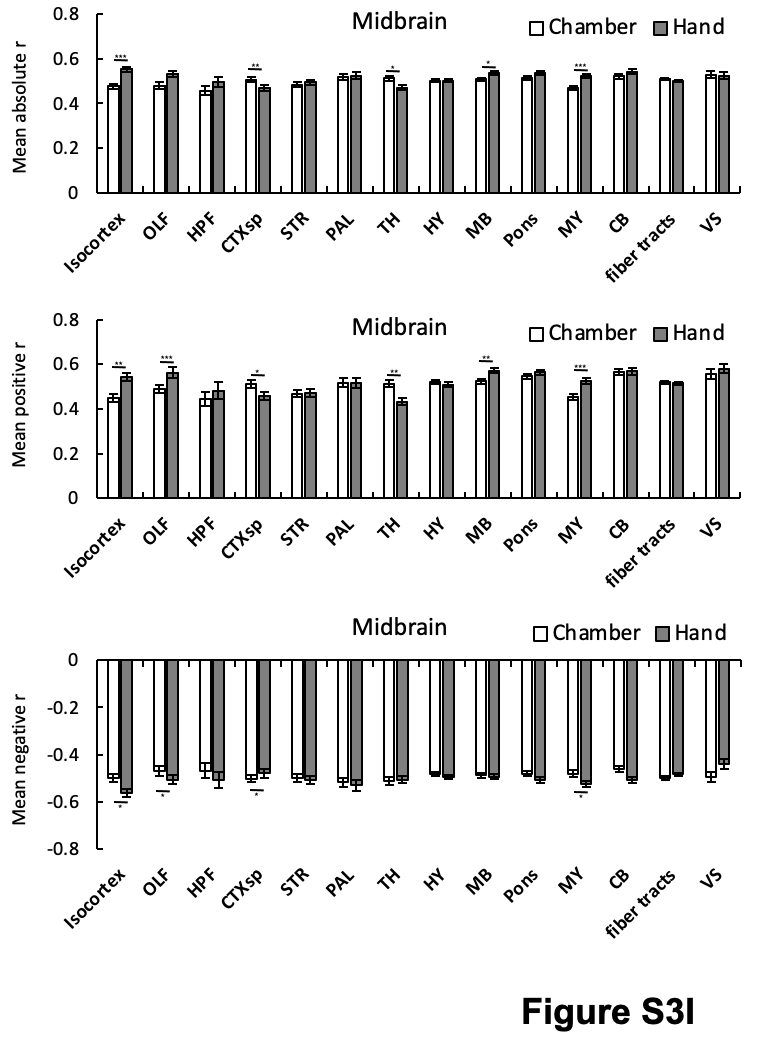


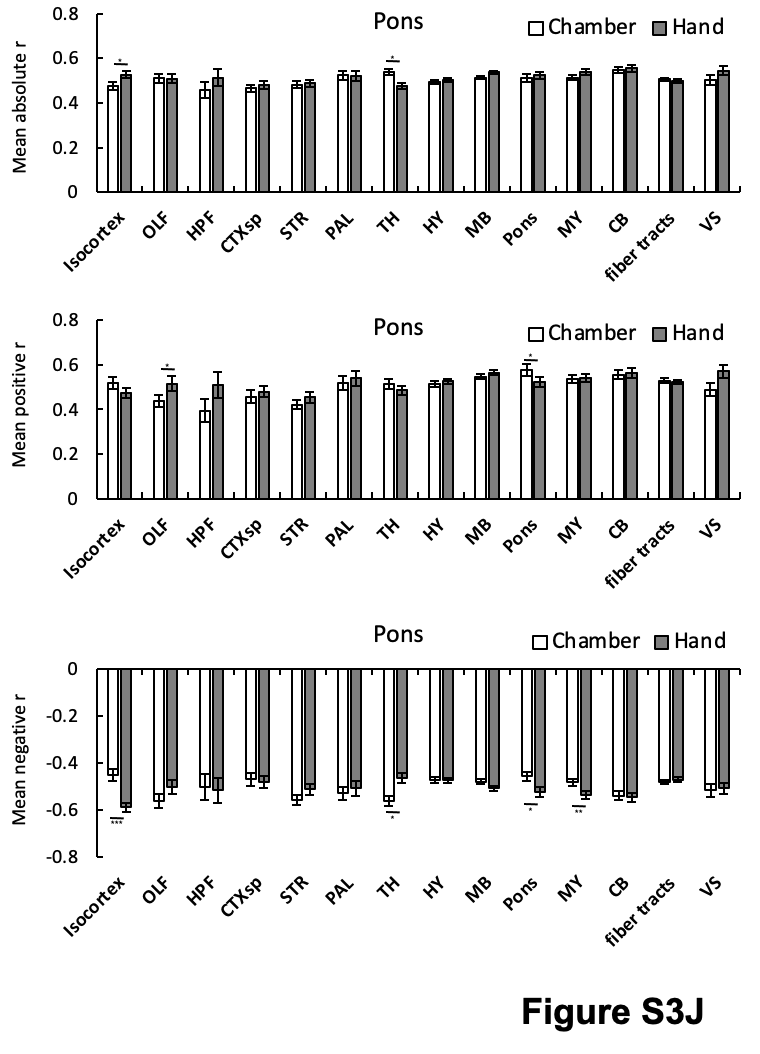


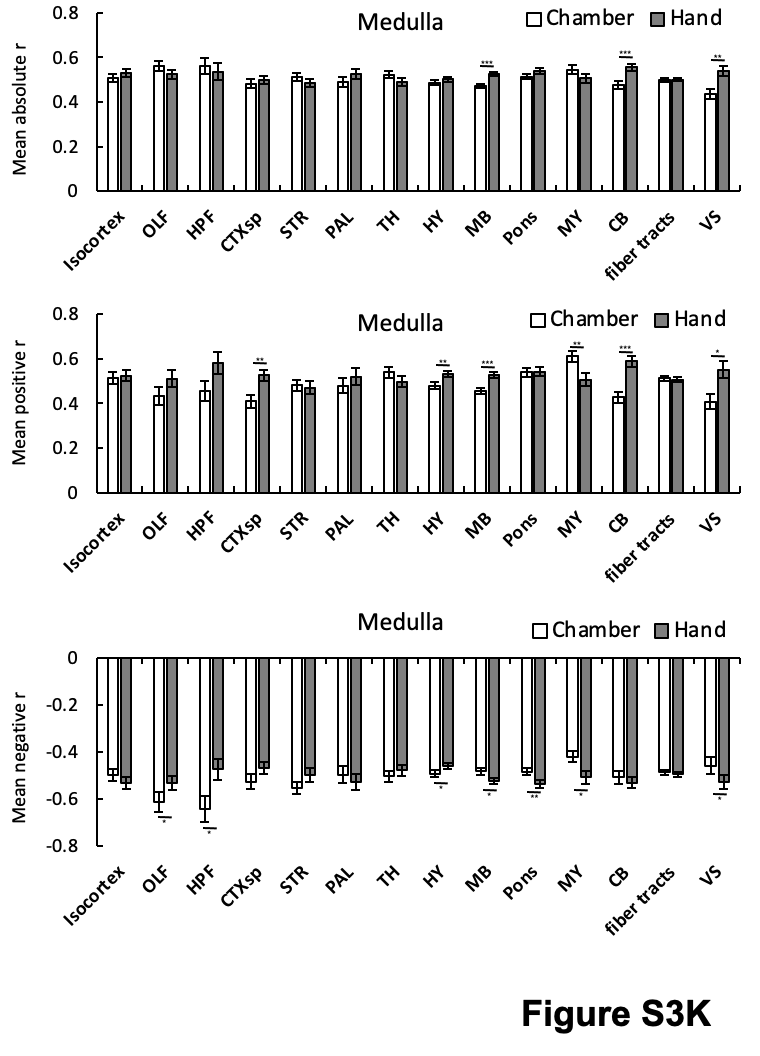


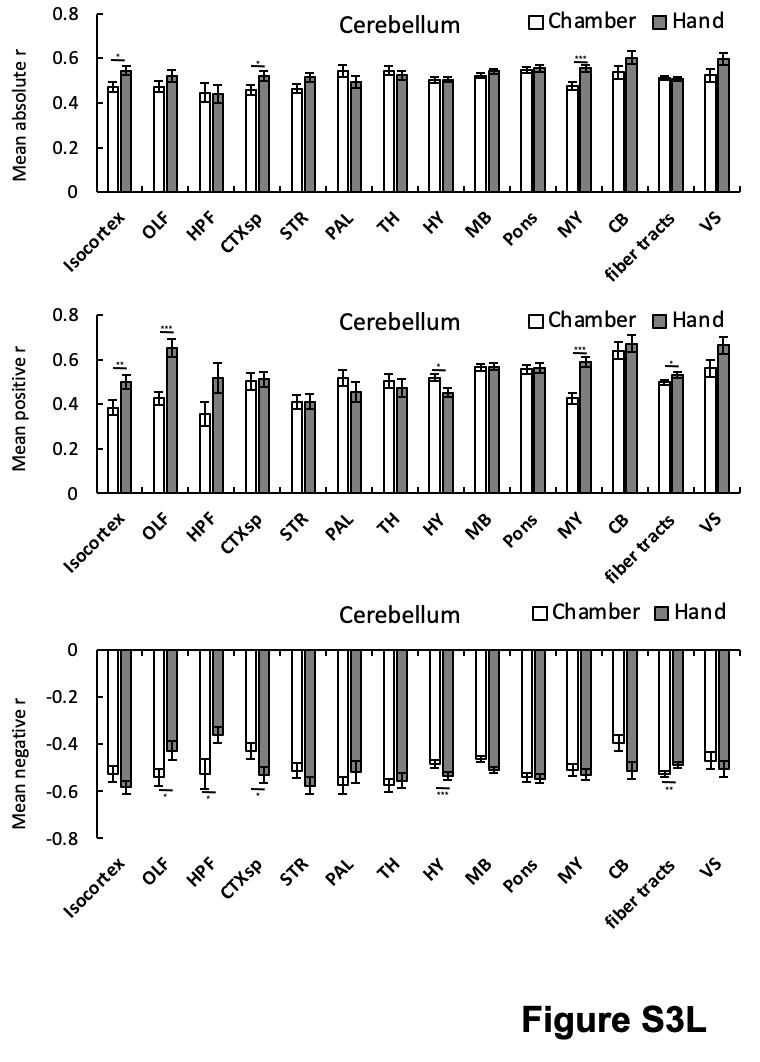


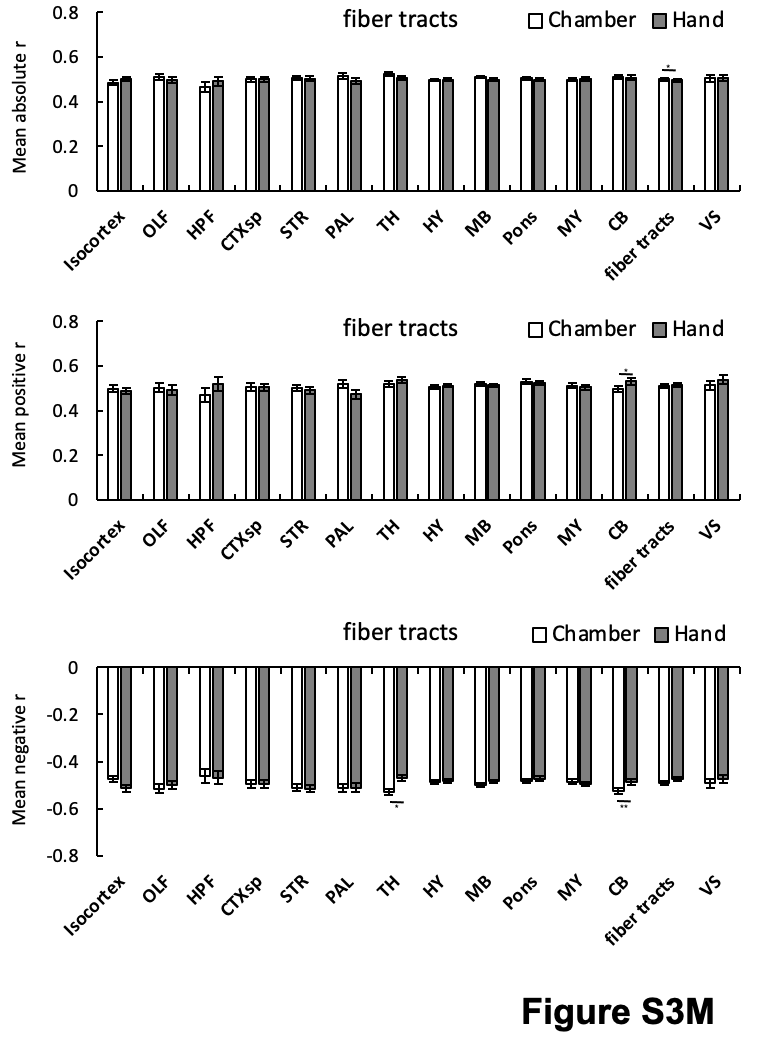


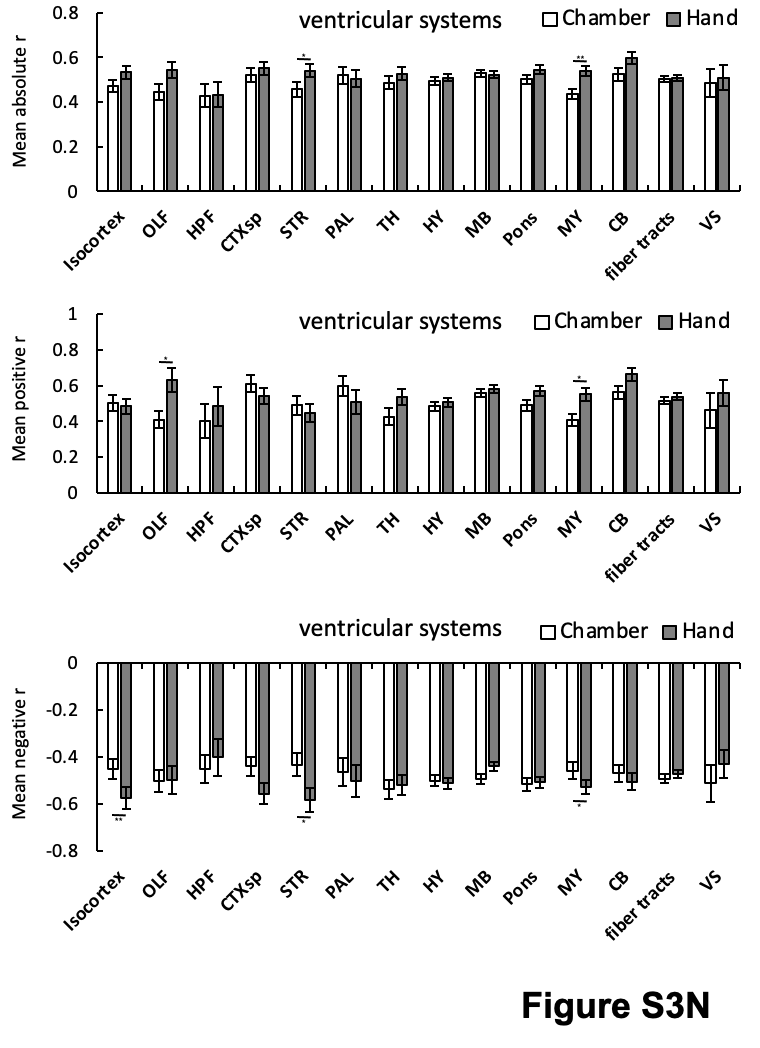


**Supplementary Figure S3** Conditional mean r changes for connectivity among brain regions due to conditions.

Three conditional mean r for connectivity among brain regions, mean absolute r (top), mean positive r (middle), and mean negative r (bottom) are shown for each 14 regions, **(A)** isocortex; **(B)** olfactory areas; **(C)** hippocampal formation; **(D)** cortical subplate; **(E)** striatum; **(F)** pallidum; **(G)** thalamus; **(H)** hypothalamus; **(I)** midbrain; **(J)** pons; **(K)** medulla; **(L)** cerebellum; **(M)** fiber tracts; and **(N)** ventricular systems. The error bars indicate SEM. *, *p* < 0.05; **, *p* < 0.01; and ***, *p* < 0.001 for the difference between gentle hand and chamber conditions by Kolmogorov-Smirnov test.

**Supplementary Table S1** Total number of cFos-TRAPed cells.

**Supplementary Table S2** Ratio of cfos-TRAPed cells in 14 brain regions and results of two-way ANOVA.

Ratio of cfos-TRAPed cells and two-way ANOVA results in the 14 regions are shown. Regions with *p* <0.05 by two-way ANOVA are shown in bold.

**Supplementary Table S3** Ratio of cFos-TRAPed cells in subdivided brain regions and results of two-way ANOVA.

| Table S3A |  |  |  |  |  |  |  |  |  |  |
| --- | --- | --- | --- | --- | --- | --- | --- | --- | --- | --- |
| Brain region | Acronym | Ratio (# of cells in each class 6 region / # of cells in each class 1-5 region) | | | | |  | p value by two-way ANOVA | | |
|  |  | *Ad lib* sleep | |  | Sleep deprivation | |  | Condition | Method | Condition x Method interaction |
|  |  | Chamber | Hand |  | Chamber | Hand |  |  |  |  |
| **Isocortex** | Isocortex |  |  |  |  |  |  |  |  |  |
| Frontal pole cerebral cortex | FRP | 0.0% | 0.0% |  | 0.0% | 0.0% |  | - | - | - |
| Somatomotor areas | MO | 11.9% | 10.9% |  | 10.8% | 11.8% |  | 0.9412 | 0.9858 | 0.4931 |
| Somatosensory areas | SS | 48.5% | 42.9% |  | 40.9% | 43.6% |  | 0.5070 | 0.7623 | 0.4721 |
| Gustatory areas | GU | 1.8% | 1.5% |  | 1.0% | 1.4% |  | 0.0905 | 0.8848 | 0.2087 |
| Visceral area | VISC | 2.0% | 1.3% |  | 1.7% | 2.2% |  | 0.5942 | 0.8992 | 0.2905 |
| Auditory areas | AUD | 5.0% | 6.6% |  | 7.8% | 4.8% |  | 0.7204 | 0.5942 | 0.1339 |
| Visual areas | VIS | 7.9% | 11.7% |  | 10.3% | 9.5% |  | 0.9726 | 0.6062 | 0.4299 |
| Anterior cingulate area | ACA | 4.0% | 4.4% |  | 4.8% | 5.0% |  | 0.5311 | 0.7892 | 0.9604 |
| Prelimbic area | PL | 0.0% | 0.0% |  | 0.1% | 0.0% |  | 0.2388 | 0.3859 | 0.3859 |
| Infralimbic area | ILA | 0.0% | 0.0% |  | 0.0% | 0.0% |  | 0.3739 | 0.3739 | 0.3739 |
| Orbital area | ORB | 0.0% | 0.0% |  | 0.0% | 0.0% |  | - | - | - |
| Agranular insular area | AI | 1.9% | 1.9% |  | 2.3% | 2.6% |  | 0.1021 | 0.4750 | 0.5604 |
| Retrosplenial area | RSP | 9.7% | 10.7% |  | 12.1% | 10.9% |  | 0.5770 | 0.9800 | 0.6326 |
| Posterior parietal association areas | PTLp | 5.4% | 5.1% |  | 5.9% | 5.7% |  | 0.4058 | 0.7732 | 0.9291 |
| Temporal association areas | TEa | 1.1% | 1.8% |  | 1.5% | 1.6% |  | 0.7024 | 0.2895 | 0.4747 |
| Perirhinal area | PERI | 0.1% | 0.1% |  | 0.1% | 0.2% |  | 0.0824 | 0.0930 | 0.2110 |
| Ectorhinal area | ECT | 0.7% | 0.9% |  | 0.8% | 0.9% |  | 0.8998 | 0.3992 | 0.7160 |

| Table S3B |  |  |  |  |  |  |  |  |  |  |
| --- | --- | --- | --- | --- | --- | --- | --- | --- | --- | --- |
| Brain region | Acronym | Ratio (# of cells in each class 6 region / # of cells in each class 1-5 region) | | | | |  | p value by two-way ANOVA | | |
|  |  | *Ad lib* sleep | |  | Sleep deprivation | |  | Condition | Method | Condition x Method interaction |
|  |  | Chamber | Hand |  | Chamber | Hand |  |  |  |  |
| **Olfactory areas** | OLF |  |  |  |  |  |  |  |  |  |
| Main olfactory bulb | MOB | 0.0% | 0.0% |  | 0.0% | 0.0% |  | - | - | - |
| Accessory olfactory bulb | AOB | 0.0% | 0.0% |  | 0.0% | 0.0% |  | - | - | - |
| Anterior olfactory nucleus | AON | 0.0% | 0.0% |  | 0.0% | 0.0% |  | - | - | - |
| Taenia tecta | TT | 0.4% | 0.2% |  | 0.5% | 1.1% |  | 0.4163 | 0.6904 | 0.5132 |
| Dorsal peduncular area | DP | 0.1% | 0.0% |  | 0.6% | 0.1% |  | 0.2420 | 0.2908 | 0.4409 |
| Piriform area | PIR | 59.8% | 59.5% |  | 60.7% | 61.8% |  | 0.4573 | 0.8566 | 0.7400 |
| Nucleus of the lateral olfactory tract | NLOT | 3.3% | 4.0% |  | 3.0% | 3.1% |  | 0.5482 | 0.6819 | 0.7786 |
| Cortical amygdalar area | COA | 23.8% | 22.8% |  | 21.5% | 21.4% |  | 0.3091 | 0.7551 | 0.7865 |
| Piriform-amygdalar area | PAA | 6.8% | 5.3% |  | 7.7% | 5.5% |  | 0.4942 | 0.0685 | 0.6455 |
| Postpiriform transition area | TR | 2.1% | 3.7% |  | 2.5% | 2.8% |  | 0.6566 | 0.1082 | 0.2544 |
| **Other olfactory areas** | Other OLF | 3.8% | 4.5% |  | 3.7% | 4.1% |  | 0.0550 | **0.0049** | 0.2572 |

| Table S3C |  |  |  |  |  |  |  |  |  |  |
| --- | --- | --- | --- | --- | --- | --- | --- | --- | --- | --- |
| Brain region | Acronym | Ratio (# of cells in each class 6 region / # of cells in each class 1-5 region) | | | | |  | p value by two-way ANOVA | | |
|  |  | *Ad lib* sleep | |  | Sleep deprivation | |  | Condition | Method | Condition x Method interaction |
|  |  | Chamber | Hand |  | Chamber | Hand |  |  |  |  |
| **Hippocampal formation** | HPF |  |  |  |  |  |  |  |  |  |
| Hippocampal region | HIP | 76.9% | 52.7% |  | 74.6% | 71.9% |  | 0.3677 | 0.1825 | 0.2682 |
| Retrohippocampal region | RHP | 21.7% | 46.3% |  | 24.4% | 27.4% |  | 0.3804 | 0.1693 | 0.2601 |
| Other hippocampal formation | Other HPF | 1.4% | 1.0% |  | 1.0% | 0.7% |  | 0.3706 | 0.3518 | 0.8210 |

| Table S3D |  |  |  |  |  |  |  |  |  |  |
| --- | --- | --- | --- | --- | --- | --- | --- | --- | --- | --- |
| Brain region | Acronym | Ratio (# of cells in each class 6 region / # of cells in each class 1-5 region) | | | | |  | p value by two-way ANOVA | | |
|  |  | *Ad lib* sleep | |  | Sleep deprivation | |  | Condition | Method | Condition x Method interaction |
|  |  | Chamber | Hand |  | Chamber | Hand |  |  |  |  |
| **Cortical subplate** | CTXsp |  |  |  |  |  |  |  |  |  |
| **Claustrum** | CLA | 2.9% | 7.4% |  | 4.0% | 4.7% |  | 0.3987 | **0.0309** | 0.0761 |
| Endopiriform nucleus dorsal part | EPd | 9.2% | 8.5% |  | 7.8% | 12.0% |  | 0.5324 | 0.3176 | 0.1804 |
| Endopiriform nucleus ventral part | EPv | 9.4% | 10.0% |  | 9.6% | 11.3% |  | 0.5135 | 0.3162 | 0.6263 |
| Lateral amygdalar nucleus | LA | 7.3% | 9.8% |  | 7.6% | 7.4% |  | 0.2716 | 0.2432 | 0.1661 |
| **Basolateral amygdalar nucleus anterior part** | BLAa | 16.3% | 15.1% |  | 15.4% | 11.7% |  | **0.0385** | **0.0256** | 0.1481 |
| Basolateral amygdalar nucleus posterior part | BLAp | 5.6% | 6.5% |  | 8.4% | 5.7% |  | 0.2769 | 0.3330 | 0.0822 |
| Basolateral amygdalar nucleus ventral part | BLAv | 5.6% | 2.5% |  | 5.1% | 4.6% |  | 0.3190 | 0.0543 | 0.1226 |
| Basomedial amygdalar nucleus anterior part | BMAa | 23.4% | 20.0% |  | 18.4% | 22.0% |  | 0.6625 | 0.9722 | 0.3326 |
| Basomedial amygdalar nucleus posterior part | BMAp | 7.9% | 7.2% |  | 9.7% | 7.1% |  | 0.6903 | 0.4286 | 0.6576 |
| Posterior amygdalar nucleus | PA | 9.0% | 8.9% |  | 8.9% | 10.2% |  | 0.6833 | 0.7100 | 0.6491 |
| Other cortical subplate | Other CTXsp | 3.4% | 4.1% |  | 5.1% | 3.2% |  | 0.6019 | 0.4850 | 0.1528 |

| Table S3E |  |  |  |  |  |  |  |  |  |  |
| --- | --- | --- | --- | --- | --- | --- | --- | --- | --- | --- |
| Brain region | Acronym | Ratio (# of cells in each class 6 region / # of cells in each class 1-5 region) | | | | |  | p value by two-way ANOVA | | |
|  |  | *Ad lib* sleep | |  | Sleep deprivation | |  | Condition | Method | Condition x Method interaction |
|  |  | Chamber | Hand |  | Chamber | Hand |  |  |  |  |
| **Striatum** | STR |  |  |  |  |  |  |  |  |  |
| **Caudoputamen** | CP | 27.5% | 28.7% |  | 31.7% | 42.0% |  | **0.0007** | **0.0033** | **0.0079** |
| **Nucleus accumbens** | ACB | 8.4% | 9.8% |  | 5.9% | 7.4% |  | **0.0012** | **0.0080** | 0.8488 |
| Fundus of striatum | FS | 0.8% | 1.1% |  | 1.3% | 0.7% |  | 0.6619 | 0.4469 | 0.0619 |
| Olfactory tubercle | OT | 5.6% | 5.6% |  | 6.1% | 7.4% |  | 0.0522 | 0.1810 | 0.1821 |
| Lateral septal nucleus | LS | 21.2% | 18.3% |  | 15.6% | 14.3% |  | 0.0542 | 0.3070 | 0.6803 |
| Septofimbrial nucleus | SF | 0.7% | 0.8% |  | 0.9% | 1.0% |  | 0.3717 | 0.5735 | 0.8810 |
| **Septohippocampal nucleus** | SH | 0.1% | 0.1% |  | 0.0% | 0.0% |  | **0.0081** | 0.6494 | 0.5938 |
| Anterior amygdalar area | AAA | 1.3% | 1.6% |  | 1.306% | 1.001% |  | 0.6189 | 0.9893 | 0.5585 |
| Bed nucleus of the accessory olfactory tract | BA | 0.0% | 0.0% |  | 0.3% | 0.1% |  | 0.2598 | 0.3963 | 0.3963 |
| Central amygdalar nucleus | CEA | 9.7% | 8.4% |  | 8.0% | 5.2% |  | 0.0520 | 0.0800 | 0.4423 |
| Intercalated amygdalar nucleus | IA | 1.5% | 1.4% |  | 2.0% | 1.0% |  | 0.7912 | 0.0595 | 0.1297 |
| Medial amygdalar nucleus | MEA | 15.9% | 15.9% |  | 16.4% | 12.5% |  | 0.3079 | 0.1950 | 0.1814 |
| **Other striatum** | Other STR | 7.5% | 8.4% |  | 10.6% | 7.3% |  | 0.1565 | 0.1081 | **0.0211** |

| Table S3F |  |  |  |  |  |  |  |  |  |  |
| --- | --- | --- | --- | --- | --- | --- | --- | --- | --- | --- |
| Brain region | Acronym | Ratio (# of cells in each class 6 region / # of cells in each class 1-5 region) | | | | |  | p value by two-way ANOVA | | |
|  |  | *Ad lib* sleep | |  | Sleep deprivation | |  | Condition | Method | Condition x Method interaction |
|  |  | Chamber | Hand |  | Chamber | Hand |  |  |  |  |
| **Pallidum** | PAL |  |  |  |  |  |  |  |  |  |
| **Globus pallidus external segment** | GPe | 9.4% | 9.0% |  | 11.5% | 7.5% |  | 0.6855 | **0.0320** | 0.0599 |
| Globus pallidus internal segment | GPi | 2.5% | 1.1% |  | 1.1% | 1.6% |  | 0.5219 | 0.4827 | 0.1964 |
| Substantia innominata | SI | 23.8% | 22.4% |  | 22.8% | 23.7% |  | 0.9566 | 0.9513 | 0.7005 |
| **Magnocellular nucleus** | MA | 2.9% | 1.5% |  | 3.2% | 2.4% |  | 0.0988 | **0.0197** | 0.3744 |
| Medial septal complex | MSC | 9.0% | 9.6% |  | 10.7% | 11.7% |  | 0.1994 | 0.5460 | 0.8612 |
| Triangular nucleus of septum | TRS | 4.0% | 3.0% |  | 1.6% | 4.9% |  | 0.8880 | 0.4509 | 0.1976 |
| Bed nuclei of the stria terminalis | BST | 38.5% | 45.9% |  | 38.2% | 39.4% |  | 0.5158 | 0.4161 | 0.5577 |
| Bed nucleus of the anterior commissure | BAC | 0.3% | 0.0% |  | 0.0% | 0.0% |  | 0.3739 | 0.3739 | 0.3739 |
| Other pallidum | Other PAL | 9.767% | 7.545% |  | 10.993% | 8.792% |  | 0.5138 | 0.2697 | 0.9954 |

| Table S3G |  |  |  |  |  |  |  |  |  |  |
| --- | --- | --- | --- | --- | --- | --- | --- | --- | --- | --- |
| Brain region | Acronym | Ratio (# of cells in each class 6 region / # of cells in each class 1-5 region) | | | | |  | p value by two-way ANOVA | | |
|  |  | *Ad lib* sleep | |  | Sleep deprivation | |  | Condition | Method | Condition x Method interaction |
|  |  | Chamber | Hand |  | Chamber | Hand |  |  |  |  |
| **Thalamus** | TH |  |  |  |  |  |  |  |  |  |
| Ventral group of the dorsal thalamus | VENT | 13.1% | 10.6% |  | 10.2% | 8.0% |  | 0.1237 | 0.1799 | 0.9437 |
| Subparafascicular nucleus | SPF | 2.2% | 2.2% |  | 2.5% | 2.7% |  | 0.5408 | 0.9444 | 0.8519 |
| Subparafascicular area | SPA | 0.5% | 2.5% |  | 0.5% | 1.4% |  | 0.5599 | 0.1812 | 0.5680 |
| Peripeduncular nucleus | PP | 0.5% | 0.2% |  | 0.2% | 0.4% |  | 0.9622 | 0.8987 | 0.1806 |
| Geniculate group dorsal thalamus | GENd | 4.3% | 3.6% |  | 3.4% | 3.9% |  | 0.4173 | 0.8853 | 0.2119 |
| Lateral group of the dorsal thalamus | LAT | 6.6% | 6.7% |  | 6.7% | 7.0% |  | 0.7565 | 0.7796 | 0.8912 |
| Anterior group of the dorsal thalamus | ATN | 6.4% | 8.8% |  | 5.9% | 10.1% |  | 0.7933 | 0.0840 | 0.5769 |
| Medial group of the dorsal thalamus | MED | 14.7% | 12.0% |  | 17.5% | 14.7% |  | 0.2466 | 0.2449 | 0.9905 |
| Midline group of the dorsal thalamus | MTN | 21.0% | 27.2% |  | 25.1% | 24.3% |  | 0.8764 | 0.4802 | 0.3788 |
| Intralaminar nuclei of the dorsal thalamus | ILM | 11.6% | 12.5% |  | 13.0% | 12.3% |  | 0.5606 | 0.8927 | 0.4940 |
| **Reticular nucleus of the thalamus** | RT | 2.7% | 1.7% |  | 1.5% | 1.2% |  | **0.0298** | 0.0747 | 0.2142 |
| Geniculate group ventral thalamus | GENv | 10.0% | 6.0% |  | 6.5% | 6.8% |  | 0.5302 | 0.4051 | 0.3406 |
| **Epithalamus** | EPI | 1.6% | 0.9% |  | 1.1% | 3.2% |  | **0.0204** | **0.0315** | **0.0038** |
| Other thalamus | Other TH | 4.9% | 5.0% |  | 6.1% | 4.1% |  | 0.8397 | 0.1690 | 0.1337 |

| Table S3H |  |  |  |  |  |  |  |  |  |  |
| --- | --- | --- | --- | --- | --- | --- | --- | --- | --- | --- |
| Brain region | Acronym | Ratio (# of cells in each class 6 region / # of cells in each class 1-5 region) | | | | |  | p value by two-way ANOVA | | |
|  |  | *Ad lib* sleep | |  | Sleep deprivation | |  | Condition | Method | Condition x Method interaction |
|  |  | Chamber | Hand |  | Chamber | Hand |  |  |  |  |
| **Hypothalamus** | HY |  |  |  |  |  |  |  |  |  |
| Supraoptic nucleus | SO | 0.4% | 0.4% |  | 0.3% | 0.4% |  | 0.7537 | 0.8641 | 0.8351 |
| Accessory supraoptic group | ASO | 0.2% | 0.3% |  | 0.2% | 0.1% |  | 0.4269 | 0.7257 | 0.4525 |
| Paraventricular hypothalamic nucleus | PVH | 2.5% | 4.2% |  | 3.1% | 1.4% |  | 0.1665 | 0.9624 | 0.0642 |
| Periventricular hypothalamic nucleus anterior part | PVa | 0.4% | 1.3% |  | 0.4% | 0.5% |  | 0.1723 | 0.1568 | 0.2014 |
| Periventricular hypothalamic nucleus intermediate part | PVi | 1.1% | 0.9% |  | 1.2% | 1.6% |  | 0.2943 | 0.7818 | 0.4150 |
| Arcuate hypothalamic nucleus | ARH | 2.7% | 3.8% |  | 2.6% | 2.9% |  | 0.1669 | 0.0943 | 0.2320 |
| Anterodorsal preoptic nucleus | ADP | 0.2% | 0.3% |  | 1.2% | 0.6% |  | 0.0953 | 0.4304 | 0.3155 |
| **Anteroventral preoptic nucleus** | AVP | 0.2% | 0.3% |  | 0.8% | 0.2% |  | 0.0918 | 0.0592 | **0.0283** |
| Anteroventral periventricular nucleus | AVPV | 0.2% | 0.2% |  | 1.7% | 0.9% |  | 0.0585 | 0.3609 | 0.3741 |
| Dorsomedial nucleus of the hypothalamus | DMH | 3.6% | 2.3% |  | 4.7% | 3.3% |  | 0.3637 | 0.2449 | 0.9921 |
| Median preoptic nucleus | MEPO | 0.2% | 0.1% |  | 0.3% | 0.2% |  | 0.2198 | 0.0905 | 0.5575 |
| **Medial preoptic area** | MPO | 3.1% | 3.8% |  | 2.7% | 4.9% |  | 0.4270 | **0.0321** | 0.1916 |
| **Vascular organ of the lamina terminalis** | OV | 0.0% | 0.0% |  | 0.1% | 0.0% |  | **0.0126** | **0.0041** | **0.0126** |
| Posterodorsal preoptic nucleus | PD | 0.0% | 0.0% |  | 0.0% | 0.0% |  | 0.8549 | 0.8549 | 0.2339 |
| Parastrial nucleus | PS | 0.6% | 0.7% |  | 0.9% | 0.4% |  | 0.8542 | 0.3921 | 0.2876 |
| Periventricular hypothalamic nucleus posterior part | PVp | 0.8% | 1.2% |  | 0.2% | 0.9% |  | 0.5935 | 0.4905 | 0.8782 |
| **Periventricular hypothalamic nucleus preoptic part** | PVpo | 1.4% | 1.8% |  | 0.6% | 0.9% |  | **0.0069** | 0.1355 | 0.7346 |
| Subparaventricular zone | SBPV | 1.5% | 1.9% |  | 1.2% | 1.2% |  | 0.3557 | 0.7080 | 0.6677 |
| Suprachiasmatic nucleus | SCH | 0.9% | 0.8% |  | 1.1% | 1.9% |  | 0.3403 | 0.6111 | 0.5350 |
| Subfornical organ | SFO | 0.6% | 0.8% |  | 0.6% | 0.0% |  | 0.0794 | 0.2953 | 0.0570 |
| **Ventromedial preoptic nucleus** | VMPO | 0.7% | 0.6% |  | 0.0% | 0.4% |  | **0.0073** | 0.2315 | 0.0544 |
| Ventrolateral preoptic nucleus | VLPO | 0.4% | 0.6% |  | 0.1% | 0.2% |  | 0.1964 | 0.6133 | 0.4834 |
| **Anterior hypothalamic nucleus** | AHN | 4.7% | 5.9% |  | 7.7% | 4.3% |  | 0.1290 | **0.0353** | **0.0028** |
| Lateral mammillary nucleus | LM | 0. 1% | 0.3% |  | 0.1% | 0.2% |  | 0.8762 | 0.3586 | 0.7617 |
| Medial mammillary nucleus | MM | 1.5% | 2.0% |  | 2.9% | 2.8% |  | 0.0539 | 0.6192 | 0.4943 |
| Supramammillary nucleus | SUM | 2.5% | 2.1% |  | 1.5% | 2.2% |  | 0.1308 | 0.6812 | 0.1038 |
| Tuberomammillary nucleus | TM | 0.7% | 2.1% |  | 0.7% | 0.4% |  | 0.2081 | 0.4280 | 0.2090 |
| Medial preoptic nucleus | MPN | 2.6% | 3.2% |  | 2.6% | 2.0% |  | 0.2203 | 0.9998 | 0.2019 |
| Dorsal premammillary nucleus | PMd | 1.0% | 1.0% |  | 0.4% | 0.8% |  | 0.2437 | 0.5519 | 0.4774 |
| Ventral premammillary nucleus | PMv | 1.6% | 2.8% |  | 0.9% | 1.6% |  | 0.2730 | 0.2849 | 0.7508 |
| Paraventricular hypothalamic nucleus descending division | PVHd | 2.1% | 1.2% |  | 2.0% | 1.5% |  | 0.8898 | 0.2953 | 0.7670 |
| Ventromedial hypothalamic nucleus | VMH | 3.9% | 2.7% |  | 2.7% | 4.6% |  | 0.7490 | 0.7149 | 0.1978 |
| Posterior hypothalamic nucleus | PH | 9.3% | 7.0% |  | 8.5% | 9.5% |  | 0.5052 | 0.5632 | 0.2080 |
| **Lateral hypothalamic area** | LHA | 9.2% | 9.1% |  | 10.7% | 10.8% |  | **0.0218** | 0.9711 | 0.8070 |
| Lateral preoptic area | LPO | 2.4% | 3.1% |  | 1.8% | 2.7% |  | 0.5284 | 0.3157 | 0.9174 |
| **Preparasubthalamic nucleus** | PST | 0.5% | 0.0% |  | 0.2% | 0.0% |  | 0.0910 | **0.0118** | 0.0910 |
| Parasubthalamic nucleus | PSTN | 2.0% | 2.4% |  | 2.0% | 1.8% |  | 0.7108 | 0.8631 | 0.7010 |
| Perifornical nucleus | PeF | 1.0% | 0.8% |  | 1.1% | 1.0% |  | 0.5223 | 0.6647 | 0.9847 |
| Retrochiasmatic area | RCH | 1.0% | 1.1% |  | 0.7% | 1.1% |  | 0.6069 | 0.3133 | 0.6995 |
| Subthalamic nucleus | STN | 0.8% | 0.4% |  | 0.4% | 0.4% |  | 0.2778 | 0.2505 | 0.2949 |
| **Tuberal nucleus** | TU | 3.8% | 3.3% |  | 3.7% | 2.1% |  | 0.1289 | **0.0279** | 0.1453 |
| Zona incerta | ZI | 6.9% | 4.1% |  | 6.2% | 4.8% |  | 0.9803 | 0.0568 | 0.4202 |
| Fields of Forel | FF | 1.0% | 0.9% |  | 0.6% | 1.2% |  | 0.8974 | 0.5089 | 0.4020 |
| Median eminence | ME | 0.9% | 0.5% |  | 0.4% | 0.5% |  | 0.6290 | 0.7000 | 0.5794 |
| Other hypothalamus | Other HY | 18.7% | 18.1% |  | 17.9% | 20.9% |  | 0.6652 | 0.6078 | 0.4534 |

| Table S3I |  |  | |  |  |  | |  |  |  |  |  |
| --- | --- | --- | --- | --- | --- | --- | --- | --- | --- | --- | --- | --- |
| Brain region | Acronym | | Ratio (# of cells in each class 6 region / # of cells in each class 1-5 region) | | | | | |  | p value by two-way ANOVA | | |
|  |  |  | *Ad lib* sleep | |  | Sleep deprivation | | |  | Condition | Method | Condition x Method interaction |
|  |  |  | Chamber | Hand |  | Chamber | Hand | |  |  |  |  |
| **Midbrain** | MB | |  |  |  |  |  | |  |  |  |  |
| **Superior colliculus optic layer** | SCop | | 5.8% | 4.0% |  | 3.9% | 5.1% | |  | 0.4099 | 0.5732 | **0.0332** |
| Superior colliculus superficial gray layer | SCsg | | 10.2% | 11.1% |  | 7.8% | 12.0% | |  | 0.6569 | 0.1894 | 0.3603 |
| Superior colliculus zonal layer | SCzo | | 1.8% | 1.7% |  | 1.7% | 1.7% | |  | 0.8993 | 0.9032 | 0.9290 |
| Inferior colliculus central nucleus | ICc | | 3.4% | 5.8% |  | 3.9% | 6.9% | |  | 0.5635 | 0.1074 | 0.8322 |
| **Inferior colliculus dorsal nucleus** | ICd | | 2.6% | 4.5% |  | 2.3% | 5.3% | |  | 0.6170 | **0.0067** | 0.2978 |
| Inferior colliculus external nucleus | ICe | | 5.6% | 5.1% |  | 5.2% | 2.9% | |  | 0.1630 | 0.1372 | 0.3034 |
| Nucleus of the brachium of the inferior colliculus | NB | | 0.1% | 0.1% |  | 0.1% | 0.0% | |  | 0.2587 | 0.8362 | 0.6588 |
| Nucleus sagulum | SAG | | 0.0% | 0.0% |  | 0.1% | 0.0% | |  | 0.3386 | 0.2626 | 0.7376 |
| **Parabigeminal nucleus** | PBG | | 0.0% | 0.0% |  | 0.0% | 0.0% | |  | **0.0272** | 0.1277 | 0.1277 |
| Midbrain trigeminal nucleus | MEV | | 0.1% | 0.0% |  | 0.0% | 0.0% | |  | 0.9424 | 0.4993 | 0.6095 |
| Subcommissural organ | SCO | | 0.0% | 0.0% |  | 0.0% | 0.0% | |  | 0.9741 | 0.2303 | 0.9741 |
| Substantia nigra reticular part | SNr | | 0.5% | 1.3% |  | 0.5% | 1.5% | |  | 0.7075 | 0.0528 | 0.7006 |
| **Ventral tegmental area** | VTA | | 0.4% | 0.4% |  | 0.7% | 0.8% | |  | **0.0461** | 0.7514 | 0.8823 |
| Paranigral nucleus | PN | | 0.0% | 0.1% |  | 0.0% | 0.0% | |  | 0.5549 | 0.2421 | 0.6579 |
| Midbrain reticular nucleus retrorubral area | RR | | 0.2% | 0.1% |  | 0.0% | 0.2% | |  | 0.7901 | 0.5128 | 0.1907 |
| Midbrain reticular nucleus | MRN | | 8.3% | 7.5% |  | 8.7% | 6.8% | |  | 0.8334 | 0.0651 | 0.3808 |
| Superior colliculus motor related deep gray layer | SCdg | | 3.2% | 2.5% |  | 3.8% | 2.1% | |  | 0.8534 | 0.0590 | 0.3877 |
| Superior colliculus motor related deep white layer | SCdw | | 0.6% | 0.7% |  | 0.8% | 0.5% | |  | 0.9360 | 0.5334 | 0.3480 |
| **Superior colliculus motor related intermediate white layer** | SCiw | | 8.0% | 5.9% |  | 8.2% | 5.9% | |  | 0.9097 | **0.0163** | 0.8245 |
| **Superior colliculus motor related intermediate gray layer** | SCig | | 10.6% | 7.8% |  | 8.5% | 7.8% | |  | 0.1142 | **0.0282** | 0.1181 |
| Periaqueductal gray | PAG | | 15.7% | 14.1% |  | 17.1% | 13.7% | |  | 0.7079 | 0.1194 | 0.5179 |
| Precommissural nucleus | PRC | | 0.4% | 0.7% |  | 0.4% | 0.9% | |  | 0.6974 | 0.1290 | 0.6485 |
| Interstitial nucleus of Cajal | INC | | 0.1% | 0.1% |  | 0.2% | 0.1% | |  | 0.5922 | 0.3717 | 0.5441 |
| Nucleus of Darkschewitsch | ND | | 0.2% | 0.2% |  | 0.2% | 0.2% | |  | 0.4169 | 0.6124 | 0.5121 |
| Supraoculomotor periaqueductal gray | Su3 | | 0.2% | 0.3% |  | 0.2% | 0.3% | |  | 0.9296 | 0.3289 | 0.6865 |
| Anterior pretectal nucleus | APN | | 3.5% | 3.1% |  | 3.6% | 3.0% | |  | 0.9665 | 0.5590 | 0.8530 |
| Medial pretectal area | MPT | | 0.2% | 0.4% |  | 0.3% | 0.4% | |  | 0.7902 | 0.4748 | 0.8978 |
| Nucleus of the optic tract | NOT | | 1.0% | 1.4% |  | 1.0% | 1.7% | |  | 0.7478 | 0.3039 | 0.8238 |
| Nucleus of the posterior commissure | NPC | | 0.5% | 0.9% |  | 1.1% | 0.9% | |  | 0.3960 | 0.7580 | 0.3823 |
| Olivary pretectal nucleus | OP | | 0.3% | 0.2% |  | 0.1% | 0.1% | |  | 0.0908 | 0.7263 | 0.6690 |
| Posterior pretectal nucleus | PPT | | 0.3% | 0.7% |  | 1.0% | 0.5% | |  | 0.3399 | 0.7630 | 0.1639 |
| Retroparafascicular nucleus | RPF | | 0.1% | 0.2% |  | 0.1% | 0.1% | |  | 0.8541 | 0.9519 | 0.9028 |
| Cuneiform nucleus | CUN | | 0.2% | 0.5% |  | 0.4% | 0.3% | |  | 0.9916 | 0.5773 | 0.3312 |
| Red nucleus | RN | | 0.6% | 0.8% |  | 0.3% | 0.6% | |  | 0.1275 | 0.1234 | 0.8164 |
| Oculomotor nucleus | III | | 0.1% | 0.1% |  | 0.1% | 0.1% | |  | 0.6670 | 0.6199 | 0.6621 |
| Medial accesory oculomotor nucleus | MA3 | | 0.1% | 0.0% |  | 0.0% | 0.0% | |  | 0.5578 | 0.5011 | 0.5609 |
| Edinger-Westphal nucleus | EW | | 0.1% | 0.1% |  | 0.1% | 0.1% | |  | 0.7398 | 0.3416 | 0.6232 |
| Trochlear nucleus | IV | | 0.0% | 0.0% |  | 0.0% | 0.0% | |  | 0.5124 | 0.7646 | 0.5379 |
| Paratrochlear nucleus | Pa4 | | 0.0% | 0.0% |  | 0.0% | 0.0% | |  | 0.3820 | 0.9391 | 0.7787 |
| Ventral tegmental nucleus | VTN | | 0.0% | 0.0% |  | 0.0% | 0.0% | |  | 0.3952 | 0.3089 | 0.7471 |
| Anterior tegmental nucleus | AT | | 0.0% | 0.1% |  | 0.1% | 0.0% | |  | 0.8664 | 0.7654 | 0.6801 |
| Lateral terminal nucleus of the accessory optic tract | LT | | 0.0% | 0.0% |  | 0.1% | 0.0% | |  | 0.5907 | 0.4788 | 0.1759 |
| Dorsal terminal nucleus of the accessory optic tract | DT | | 0.0% | 0.0% |  | 0.0% | 0.0% | |  | 0.4546 | 0.2342 | 0.9752 |
| **Medial terminal nucleus of the accessory optic tract** | MT | | 0.0% | 0.1% |  | 0.2% | 0.0% | |  | 0.6909 | 0.1292 | **0.0144** |
| Substantia nigra compact part | SNc | | 0.1% | 0.0% |  | 0.2% | 0.1% | |  | 0.2509 | 0.4555 | 0.8540 |
| Pedunculopontine nucleus | PPN | | 0.8% | 1.0% |  | 1.9% | 0.6% | |  | 0.4440 | 0.3321 | 0.1999 |
| Interfascicular nucleus raphe | IF | | 0.2% | 0.2% |  | 0.2% | 0.2% | |  | 0.8919 | 0.8179 | 0.9595 |
| **Interpeduncular nucleus** | IPN | | 0.4% | 0.6% |  | 0.2% | 1.2% | |  | 0.1336 | **0.0064** | **0.0247** |
| **Rostral linear nucleus raphe** | RL | | 0.3% | 0.2% |  | 0.2% | 0.1% | |  | **0.0297** | 0.0628 | 0.3605 |
| Central linear nucleus raphe | CLI | | 0.1% | 0.2% |  | 0.1% | 0.1% | |  | 0.5207 | 0.5434 | 0.5651 |
| Dorsal nucleus raphe | DR | | 0.4% | 0.5% |  | 0.3% | 0.6% | |  | 0.9181 | 0.2657 | 0.8931 |
| Other midbrain | Other MB | | 12.4% | 14.5% |  | 13.8% | 14.3% | |  | 0.5325 | 0.2436 | 0.4667 |

| Table S3J |  |  |  |  |  |  |  |  |  |  |
| --- | --- | --- | --- | --- | --- | --- | --- | --- | --- | --- |
| Brain region | Acronym | Ratio (# of cells in each class 6 region / # of cells in each class 1-5 region) | | | | |  | p value by two-way ANOVA | | |
|  |  | *Ad lib* sleep | |  | Sleep deprivation | |  | Condition | Method | Condition x Method interaction |
|  |  | Chamber | Hand |  | Chamber | Hand |  |  |  |  |
| **Pons** | Pons |  |  |  |  |  |  |  |  |  |
| Nucleus of the lateral lemniscus | NLL | 1.1% | 1.2% |  | 1.6% | 0.8% |  | 0.8929 | 0.5702 | 0.4966 |
| Principal sensory nucleus of the trigeminal | PSV | 4.0% | 3.4% |  | 5.8% | 3.3% |  | 0.2022 | 0.0517 | 0.1698 |
| Parabrachial nucleus | PB | 8.9% | 9.6% |  | 9.9% | 6.4% |  | 0.2521 | 0.1534 | 0.0626 |
| Superior olivary complex | SOC | 1.1% | 1.8% |  | 2.5% | 1.5% |  | 0.3915 | 0.7626 | 0.2131 |
| Barrington's nucleus | B | 0.3% | 0.4% |  | 0.1% | 0.0% |  | 0.1235 | 0.9700 | 0.6703 |
| Dorsal tegmental nucleus | DTN | 0.2% | 0.4% |  | 0.5% | 0.7% |  | 0.0924 | 0.3424 | 0.9346 |
| Posterodorsal tegmental nucleus | PDTg | 0.1% | 0.2% |  | 0.2% | 0.1% |  | 0.8272 | 0.8631 | 0.3215 |
| Pontine central gray | PCG | 3.3% | 3.7% |  | 3.7% | 2.8% |  | 0.8657 | 0.8445 | 0.5965 |
| **Pontine gray** | PG | 35.9% | 27.6% |  | 23.4% | 35.3% |  | 0.5418 | 0.6569 | **0.0492** |
| Pontine reticular nucleus caudal part | PRNc | 3.9% | 2.7% |  | 5.4% | 2.7% |  | 0.5780 | 0.2053 | 0.5737 |
| Supragenual nucleus | SG | 0.0% | 0.1% |  | 0.0% | 0.1% |  | 0.8152 | 0.1845 | 0.8310 |
| Supratrigeminal nucleus | SUT | 2.1% | 1.6% |  | 1.8% | 0.9% |  | 0.1791 | 0.0644 | 0.5979 |
| Tegmental reticular nucleus | TRN | 10.3% | 10.7% |  | 12.6% | 9.8% |  | 0.6527 | 0.4358 | 0.3056 |
| Motor nucleus of trigeminal | V | 0.9% | 0.8% |  | 0.7% | 0.9% |  | 0.8364 | 0.8420 | 0.5450 |
| Peritrigeminal zone | P5 | 0.6% | 0.4% |  | 0.7% | 0.2% |  | 0.6790 | 0.2021 | 0.5266 |
| Accessory trigeminal nucleus | Acs5 | 0.1% | 0.0% |  | 0.0% | 0.0% |  | 0.3739 | 0.3739 | 0.3739 |
| Parvicellular motor 5 nucleus | PC5 | 0.3% | 0.1% |  | 0.1% | 0.2% |  | 0.9467 | 0.7582 | 0.3515 |
| Intertrigeminal nucleus | I5 | 0.4% | 0.1% |  | 0.1% | 0.4% |  | 0.8591 | 0.9219 | 0.0884 |
| Superior central nucleus raphe | CS | 2.5% | 3.0% |  | 3.7% | 2.9% |  | 0.4518 | 0.7928 | 0.3815 |
| Locus ceruleus | LC | 0.2% | 0.1% |  | 0.1% | 0.0% |  | 0.4153 | 0.6084 | 0.7246 |
| Laterodorsal tegmental nucleus | LDT | 1.4% | 1.5% |  | 1.7% | 1.8% |  | 0.6455 | 0.9053 | 0.9275 |
| Nucleus incertus | NI | 1.2% | 1.0% |  | 0.6% | 0.6% |  | 0.3127 | 0.7615 | 0.8272 |
| **Pontine reticular nucleus** | PRNr | 4.1% | 3.6% |  | 6.6% | 2.7% |  | 0.3591 | **0.0458** | 0.0819 |
| Nucleus raphe pontis | RPO | 0.4% | 0.3% |  | 0.4% | 0.2% |  | 0.8671 | 0.3976 | 0.6050 |
| Subceruleus nucleus | SLC | 0.2% | 0.2% |  | 0.1% | 0.0% |  | 0.2633 | 0.9254 | 0.6639 |
| Sublaterodorsal nucleus | SLD | 0.5% | 1.1% |  | 0.1% | 0.1% |  | 0.2584 | 0.5452 | 0.5761 |
| **Other pons** | Other pons | 16.0% | 24.5% |  | 17.4% | 25.6% |  | 0.5952 | **0.0164** | 0.9386 |

| Table S3K |  |  |  |  |  |  |  |  |  |  |
| --- | --- | --- | --- | --- | --- | --- | --- | --- | --- | --- |
| Brain region | Acronym | Ratio (# of cells in each class 6 region / # of cells in each class 1-5 region) | | | | |  | p value by two-way ANOVA | | |
|  |  | *Ad lib* sleep | |  | Sleep deprivation | |  | Condition | Method | Condition x Method interaction |
|  |  | Chamber | Hand |  | Chamber | Hand |  |  |  |  |
| **Medulla** | MY |  |  |  |  |  |  |  |  |  |
| Area postrema | AP | 0.0% | 0.0% |  | 0.0% | 0.0% |  | - | - | - |
| Cochlear nuclei | CN | 9.4% | 18.0% |  | 16.1% | 20.4% |  | 0.1884 | 0.0856 | 0.4830 |
| Dorsal column nuclei | DCN | 0.0% | 0.0% |  | 0.0% | 0.0% |  | - | - | - |
| External cuneate nucleus | ECU | 0.0% | 0.0% |  | 0.0% | 0.0% |  | - | - | - |
| Nucleus of the trapezoid body | NTB | 2.6% | 2.5% |  | 2.3% | 2.9% |  | 0.9845 | 0.8887 | 0.8634 |
| Nucleus of the solitary tract | NTS | 0.4% | 0.5% |  | 0.3% | 1.6% |  | 0.3409 | 0.2371 | 0.3187 |
| Spinal nucleus of the trigeminal caudal part | SPVC | 0.0% | 0.0% |  | 0.0% | 0.0% |  | - | - | - |
| Spinal nucleus of the trigeminal interpolar part | SPVI | 0.0% | 0.0% |  | 0.0% | 0.0% |  | - | - | - |
| Spinal nucleus of the trigeminal oral part | SPVO | 10.5% | 6.3% |  | 7.4% | 8.3% |  | 0.7810 | 0.4004 | 0.2210 |
| Paratrigeminal nucleus | Pa5 | 0.0% | 0.0% |  | 0.0% | 0.0% |  | - | - | - |
| Abducens nucleus | VI | 0.3% | 0.3% |  | 0.0% | 0.2% |  | 0.3478 | 0.5831 | 0.7108 |
| **Facial motor nucleus** | VII | 6.4% | 6.1% |  | 4.5% | 1.4% |  | **0.0410** | 0.1988 | 0.2892 |
| Accessory facial motor nucleus | ACVII | 0.1% | 0.2% |  | 0.1% | 0.0% |  | 0.3799 | 0.8911 | 0.2449 |
| Nucleus ambiguus | AMB | 0.0% | 0.0% |  | 0.0% | 0.0% |  | - | - | - |
| Dorsal motor nucleus of the vagus nerve | DMX | 0.0% | 0.0% |  | 0.0% | 0.0% |  | - | - | - |
| Gigantocellular reticular nucleus | GRN | 6.6% | 6.7% |  | 8.7% | 4.0% |  | 0.8685 | 0.2292 | 0.2221 |
| Infracerebellar nucleus | ICB | 0.0% | 0.00% |  | 0.00% | 0.00% |  | - | - | - |
| Inferior olivary complex | IO | 0.0% | 0.1% |  | 0.0% | 0.0% |  | 0.3739 | 0.3739 | 0.3739 |
| Intermediate reticular nucleus | IRN | 14.3% | 12.9% |  | 10.6% | 9.7% |  | 0.2488 | 0.6814 | 0.9393 |
| Inferior salivatory nucleus | ISN | 0.2% | 0.2% |  | 0.5% | 0.0% |  | 0.6978 | 0.2205 | 0.1556 |
| Linear nucleus of the medulla | LIN | 0.0% | 0.0% |  | 0.0% | 0.0% |  | - | - | - |
| Lateral reticular nucleus | LRN | 0.0% | 0.0% |  | 0.0% | 0.0% |  | - | - | - |
| **Magnocellular reticular nucleus** | MARN | 1.6% | 2.1% |  | 2.3% | 5.0% |  | **0.0255** | **0.0349** | 0.0962 |
| Medullary reticular nucleus | MDRN | 0.0% | 0.0% |  | 0.0% | 0.0% |  | - | - | - |
| Parvicellular reticular nucleus | PARN | 18.8% | 17.2% |  | 17.8% | 13.9% |  | 0.2267 | 0.1360 | 0.4733 |
| Parasolitary nucleus | PAS | 0.0% | 0.0% |  | 0.0% | 0.0% |  | - | - | - |
| Paragigantocellular reticular nucleus | PGRN | 1.1% | 1.4% |  | 2.6% | 2.3% |  | 0.1604 | 0.9988 | 0.7091 |
| Perihypoglossal nuclei | PHY | 0.3% | 0.4% |  | 0.8% | 0.4% |  | 0.3350 | 0.6391 | 0.3026 |
| Parapyramidal nucleus | PPY | 1.0% | 0.6% |  | 0.9% | 0.9% |  | 0.6957 | 0.4990 | 0.4983 |
| **Vestibular nuclei** | VNC | 12.2% | 8.5% |  | 5.9% | 7.9% |  | **0.0358** | 0.5045 | 0.0634 |
| Nucleus x | x | 0.0% | 0.0% |  | 0.0% | 0.1% |  | 0.3739 | 0.3739 | 0.3739 |
| Hypoglossal nucleus | XII | 0.0% | 0.0% |  | 0.0% | 0.0% |  | - | - | - |
| Nucleus y | y | 0.2% | 0.1% |  | 0.0% | 0.2% |  | 0.7962 | 0.5484 | 0.4832 |
| **Nucleus raphe magnus** | RM | 1.2% | 0.7% |  | 1.6% | 0.1% |  | 0.5941 | **0.0091** | 0.0645 |
| Nucleus raphe pallidus | RPA | 0.4% | 0.3% |  | 0.6% | 0.6% |  | 0.5208 | 0.8326 | 0.8662 |
| Nucleus raphe obscurus | RO | 0.0% | 0.0% |  | 0.0% | 1.3% |  | 0.3739 | 0.3739 | 0.3739 |
| Other medulla | Other MY | 12.7% | 14.9% |  | 16.9% | 19.0% |  | 0.2896 | 0.5695 | 0.9867 |

| Table S3L |  |  |  |  |  |  |  |  |  |  |
| --- | --- | --- | --- | --- | --- | --- | --- | --- | --- | --- |
| Brain region | Acronym | Ratio (# of cells in each class 6 region / # of cells in each class 1-5 region) | | | | |  | p value by two-way ANOVA | | |
|  |  | *Ad lib* sleep | |  | Sleep deprivation | |  | Condition | Method | Condition x Method interaction |
|  |  | Chamber | Hand |  | Chamber | Hand |  |  |  |  |
| **Cerebellum** | CB |  |  |  |  |  |  |  |  |  |
| Lingula (I) | LING | 0.3% | 0.2% |  | 0.4% | 0.5% |  | 0.5511 | 0.9815 | 0.6514 |
| Lobule II | CENT2 | 12.3% | 10.2% |  | 15.8% | 6.4% |  | 0.9495 | 0.1085 | 0.2620 |
| **Lobule III** | CENT3 | 15.6% | 9.3% |  | 20.0% | 8.4% |  | 0.5045 | **0.0209** | 0.3401 |
| Lobules IV-V | CUL4 5 | 14.7% | 14.2% |  | 13.0% | 18.0% |  | 0.7188 | 0.4626 | 0.3745 |
| Declive (VI) | DEC | 0.0% | 0.0% |  | 0.0% | 0.0% |  | - | - | - |
| Folium-tuber vermis (VII) | FOTU | 0.0% | 0.0% |  | 0.0% | 0.0% |  | - | - | - |
| Pyramus (VIII) | PYR | 0.0% | 0.0% |  | 0.0% | 0.0% |  | - | - | - |
| Uvula (IX) | UVU | 0.0% | 0.0% |  | 0.0% | 0.0% |  | - | - | - |
| Nodulus (X) | NOD | 0.0% | 0.0% |  | 0.0% | 0.0% |  | - | - | - |
| Simple lobule | SIM | 21.1% | 20.0% |  | 20.5% | 23.5% |  | 0.4740 | 0.6297 | 0.3322 |
| Crus 1 | ANcr1 | 12.9% | 18.2% |  | 12.6% | 16.7% |  | 0.6493 | 0.0554 | 0.7355 |
| Crus 2 | ANcr2 | 0.5% | 0.7% |  | 0.7% | 2.8% |  | 0.1439 | 0.1300 | 0.1999 |
| Paramedian lobule | PRM | 0.1% | 0.3% |  | 0.1% | 1.2% |  | 0.4032 | 0.2340 | 0.3922 |
| Copula pyramidis | COPY | 0.4% | 0.9% |  | 0.8% | 0.3% |  | 0.8039 | 0.9633 | 0.3205 |
| Paraflocculus | PFL | 6.7% | 8.6% |  | 4.0% | 6.2% |  | 0.1721 | 0.2611 | 0.9170 |
| Flocculus | FL | 9.9% | 13.8% |  | 7.7% | 11.1% |  | 0.5425 | 0.3735 | 0.9505 |
| Fastigial nucleus | FN | 0.1% | 0.1% |  | 0.0% | 0.0% |  | 0.2308 | 0.9404 | 0.9404 |
| Interposed nucleus | IP | 0.2% | 0.4% |  | 0.3% | 1.1% |  | 0.5845 | 0.4436 | 0.6390 |
| Dentate nucleus | DN | 2.1% | 0.9% |  | 1.2% | 0.6% |  | 0.6143 | 0.4521 | 0.8332 |
| Vestibulocerebellar nucleus | VeCB | 0.1% | 0.1% |  | 0.2% | 0.0% |  | 0.8488 | 0.3543 | 0.2486 |
| Other cerebellum | Other CB | 2.9% | 2.0% |  | 2.7% | 3.3% |  | 0.6487 | 0.9093 | 0.5496 |

| Table S3M |  |  |  |  |  |  |  |  |  |  |
| --- | --- | --- | --- | --- | --- | --- | --- | --- | --- | --- |
| Brain region | Acronym | Ratio (# of cells in each class 6 region / # of cells in each class 1-5 region) | | | | |  | p value by two-way ANOVA | | |
|  |  | *Ad lib* sleep | |  | Sleep deprivation | |  | Condition | Method | Condition x Method interaction |
|  |  | Chamber | Hand |  | Chamber | Hand |  |  |  |  |
| **fiber tracts** | fiber tracts |  |  |  |  |  |  |  |  |  |
| vomeronasal nerve | von | 0.0% | 0.0% |  | 0.0% | 0.0% |  | - | - | - |
| olfactory nerve layer of main olfactory bulb | onl | 0.0% | 0.0% |  | 0.0% | 0.0% |  | - | - | - |
| lateral olfactory tract body | lot | 1.8% | 1.1% |  | 1.8% | 1.4% |  | 0.5155 | 0.0543 | 0.6712 |
| dorsal limb | lotd | 0.0% | 0.0% |  | 0.0% | 0.0% |  | - | - | - |
| anterior commissure olfactory limb | aco | 1.0% | 1.3% |  | 1.0% | 0.9% |  | 0.2708 | 0.9175 | 0.2470 |
| optic nerve | IIn | 0.0% | 0.0% |  | 0.0% | 0.0% |  | - | - | - |
| brachium of the superior colliculus | bsc | 1.1% | 0.8% |  | 1.3% | 0.2% |  | 0.4675 | 0.0669 | 0.1860 |
| superior colliculus commissure | csc | 0.5% | 0.5% |  | 0.0% | 0.3% |  | 0.3333 | 0.7329 | 0.6175 |
| optic chiasm | och | 1.4% | 3.9% |  | 2.2% | 1.1% |  | 0.5145 | 0.6622 | 0.2833 |
| optic tract | opt | 5.7% | 6.9% |  | 6.2% | 3.5% |  | 0.4946 | 0.7227 | 0.3783 |
| **oculomotor nerve** | IIIn | 0.2% | 0.0% |  | 0.0% | 0.0% |  | **0.0164** | 0.0548 | 0.0548 |
| medial longitudinal fascicle | mlf | 0.4% | 0.2% |  | 0.3% | 0.4% |  | 0.9546 | 0.8034 | 0.1607 |
| posterior commissure | pc | 0.4% | 0.6% |  | 0.8% | 0.2% |  | 0.8575 | 0.3860 | 0.1312 |
| trochlear nerve | IVn | 0.1% | 0.0% |  | 0.0% | 0.0% |  | 0.1928 | 0.1902 | 0.4890 |
| motor root of the trigeminal nerve | moV | 0.0% | 0.2% |  | 0.0% | 0.3% |  | 0.7302 | 0.2098 | 0.7302 |
| sensory root of the trigeminal nerve | sV | 0.5% | 0.3% |  | 0.9% | 0.6% |  | 0.5965 | 0.6455 | 0.9315 |
| spinal tract of the trigeminal nerve | sptV | 1.7% | 1.1% |  | 1.1% | 0.9% |  | 0.2266 | 0.2349 | 0.6416 |
| facial nerve | VIIn | 0.7% | 0.4% |  | 0.5% | 0.2% |  | 0.3976 | 0.2538 | 0.8010 |
| genu of the facial nerve | gVIIn | 0.1% | 0.2% |  | 0.0% | 0.0% |  | 0.2638 | 0.6057 | 0.6057 |
| vestibular nerve | vVIIIn | 0.5% | 0.3% |  | 0.2% | 0.2% |  | 0.4301 | 0.8553 | 0.7231 |
| trapezoid body | sptV | 0.7% | 0.5% |  | 0.6% | 0.8% |  | 0.8100 | 0.9909 | 0.6591 |
| dorsal acoustic stria | das | 0.0% | 0.0% |  | 0.0% | 0.0% |  | - | - | - |
| lateral lemniscus | ll | 2.9% | 1.5% |  | 3.7% | 1.2% |  | 0.8088 | 0.1054 | 0.5612 |
| inferior colliculus commissure | cic | 0.0% | 0.1% |  | 0.0% | 0.1% |  | 0.7160 | 0.2457 | 0.7160 |
| **brachium of the inferior colliculus** | bic | 0.5% | 0.9% |  | 1.9% | 0.6% |  | 0.1191 | 0.1930 | **0.0424** |
| solitary tract | ts | 0.0% | 0.0% |  | 0.0% | 0.0% |  | - | - | - |
| cuneate fascicle | cuf | 0.0% | 0.0% |  | 0.0% | 0.0% |  | - | - | - |
| **medial lemniscus** | ml | 10.6% | 5.6% |  | 5.1% | 5.5% |  | **0.0242** | **0.0424** | **0.0271** |
| cerebellar commissure | cbc | 0.0% | 0.0% |  | 0.0% | 0.0% |  | 0.5638 | 0.5860 | 0.6366 |
| superior cerebelar peduncles | scp | 3.5% | 3.0% |  | 3.2% | 2.4% |  | 0.5073 | 0.3282 | 0.8420 |
| superior cerebellar peduncle decussation | dscp | 0.1% | 0.2% |  | 0.3% | 0.3% |  | 0.2350 | 0.4886 | 0.8616 |
| uncinate fascicle | uf | 0.2% | 0.1% |  | 0.2% | 0.0% |  | 0.7698 | 0.2088 | 0.5709 |
| ventral spinocerebellar tract | sctv | 0.6% | 0.3% |  | 1.4% | 1.1% |  | 0.3488 | 0.6898 | 0.9880 |
| middle cerebellar peduncle | mcp | 1.4% | 2.3% |  | 1.4% | 3.4% |  | 0.4009 | 0.0785 | 0.4087 |
| inferior cerebellar peduncle | icp | 0.6% | 0.7% |  | 1.0% | 1.4% |  | 0.0736 | 0.4249 | 0.5359 |
| dorsal spinocerebellar tract | sctd | 0.0% | 0.0% |  | 0.0% | 0.0% |  | - | - | - |
| arbor vitae | arb | 6.4% | 4.9% |  | 5.3% | 7.5% |  | 0.5556 | 0.7769 | 0.2076 |
| supra-callosal cerebral white matter | scwm | 2.3% | 2.1% |  | 2.1% | 1.9% |  | 0.6565 | 0.6247 | 0.9381 |
| corpus callosum anterior forceps | fa | 0.3% | 0.5% |  | 0.7% | 0.2% |  | 0.8806 | 0.6829 | 0.3474 |
| external capsule | ec | 2.4% | 3.5% |  | 2.5% | 3.4% |  | 0.9188 | 0.0980 | 0.8907 |
| corpus callosum extreme capsule | ee | 0.4% | 0.2% |  | 0.4% | 0.2% |  | 0.9015 | 0.3359 | 0.9095 |
| **genu of corpus callosum** | ccg | 1.0% | 0.5% |  | 0.2% | 0.4% |  | **0.0252** | 0.2982 | 0.0792 |
| corpus callosum posterior forceps | fp | 1.8% | 5.7% |  | 2.0% | 7.1% |  | 0.7294 | 0.1004 | 0.7873 |
| corpus callosum body | ccb | 4.7% | 4.0% |  | 6.3% | 5.3% |  | 0.1012 | 0.2710 | 0.8226 |
| corpus callosum splenium | ccs | 0.8% | 1.3% |  | 1.0% | 1.9% |  | 0.3903 | 0.1278 | 0.6825 |
| **corticospinal tract** | cst | 0.0% | 1.7% |  | 0.0% | 0. % |  | 0.4308 | **0.0361** | 0.4308 |
| **internal capsule** | int | 4.6% | 2.9% |  | 4.1% | 2.9% |  | 0.6170 | **0.0364** | 0.7275 |
| cerebal peduncle | cpd | 1.2% | 1.6% |  | 1.5% | 2.8% |  | 0.2333 | 0.1705 | 0.4724 |
| pyramid | py | 0.6% | 0.9% |  | 0.9% | 1.4% |  | 0.4457 | 0.4087 | 0.8842 |
| pyramidal decussation | pyd | 0.0% | 0.0% |  | 0.0% | 0.0% |  | - | - | - |
| external medullary lamina of the thalamus | em | 0.1% | 0.2% |  | 0.2% | 0.0% |  | 0.6133 | 0.9187 | 0.1019 |
| optic radiation | or | 3.7% | 6.6% |  | 4.8% | 8.6% |  | 0.3366 | 0.0780 | 0.7808 |
| auditory radiation | ar | 0.8% | 0.8% |  | 0.9% | 1.1% |  | 0.6434 | 0.7462 | 0.8803 |
| nigrostriatal tract | nst | 1.4% | 0.9% |  | 1.1% | 0.2% |  | 0.1569 | 0.0601 | 0.3971 |
| direct tectospinal pathway | tspd | 0.0% | 0.0% |  | 0.0% | 0.0% |  | - | - | - |
| doral tegmental decussation | dtd | 0.0% | 0.0% |  | 0.0% | 0.0% |  | 0.9825 | 0.9825 | 0.2303 |
| crossed tectospinal pathway | tspc | 1.2% | 0.5% |  | 0.9% | 0.5% |  | 0.5525 | 0.0646 | 0.5524 |
| **rubrospinal tract** | rust | 1.9% | 1.0% |  | 1.3% | 0.9% |  | **0.0407** | **0.0066** | 0.0942 |
| ventral tegmental decussation | vtd | 0.3% | 0.2% |  | 0.0% | 0.2% |  | 0.1761 | 0.8618 | 0.1270 |
| amygdalar capsule | amc | 0.7% | 0.8% |  | 0.6% | 0.3% |  | 0.0573 | 0.3295 | 0.0968 |
| anterior commissure temporal limb | act | 1.0% | 0.8% |  | 0.6% | 0.5% |  | 0.0592 | 0.4083 | 0.7109 |
| cingulum bundle | cing | 2.7% | 5.3% |  | 5.2% | 3.8% |  | 0.6720 | 0.6053 | 0.1278 |
| alveus | alv | 2.3% | 1.6% |  | 1.9% | 3.6% |  | 0.1456 | 0.2863 | 0.0513 |
| dorsal fornix | df | 0.0% | 0.0% |  | 0.1% | 0.1% |  | 0.2313 | 0.9202 | 0.9202 |
| fimbria | fi | 2.1% | 2.1% |  | 2.5% | 2.0% |  | 0.7549 | 0.6006 | 0.6235 |
| medial corticohypothalamic tract | mct | 0.0% | 0.0% |  | 0.0% | 0.1% |  | 0.5242 | 0.2859 | 0.5242 |
| **columns of the fornix** | fx | 2.5% | 1.2% |  | 1.2% | 0.7% |  | **0.0000** | **0.0000** | **0.0000** |
| dorsal hippocampal commissure | dhc | 0.8% | 2.2% |  | 0.5% | 1.9% |  | 0.6323 | 0.0781 | 0.9371 |
| ventral hippocampal commissure | vhc | 0.8% | 0.3% |  | 0.2% | 0.6% |  | 0.6539 | 0.8893 | 0.3475 |
| stria terminalis | st | 1.0% | 1.0% |  | 1.1% | 1.1% |  | 0.7784 | 0.9862 | 0.9479 |
| commissural branch of stria terminalis | stc | 0.3% | 0.2% |  | 0.5% | 0.2% |  | 0.2685 | 0.1015 | 0.1923 |
| medial forebrain bundle | mfb | 0.7% | 0.5% |  | 0.4% | 0.9% |  | 0.8430 | 0.5162 | 0.2970 |
| supraoptic commissures | sup | 0.2% | 0.1% |  | 0.1% | 0.1% |  | 0.5707 | 0.3360 | 0.4692 |
| principal mammillary tract | pm | 0.1% | 0.4% |  | 0.2% | 0.2% |  | 0.7654 | 0.4065 | 0.3940 |
| mammillothalamic tract | mtt | 0.9% | 0.6% |  | 0.4% | 0.7% |  | 0.4085 | 0.9662 | 0.1395 |
| mammillotegmental tract | mtg | 0.7% | 0.6% |  | 0.2% | 0.6% |  | 0.4336 | 0.7014 | 0.3623 |
| **mammillary peduncle** | mp | 0.1% | 0.2% |  | 0.4% | 0.1% |  | 0.1547 | 0.1906 | **0.0158** |
| stria medullaris | sm | 1.2% | 1.1% |  | 1.5% | 0.7% |  | 0.7994 | 0.2259 | 0.2436 |
| fasciculus retroflexus | fr | 0.8% | 1.3% |  | 0.6% | 1.7% |  | 0.8095 | 0.1482 | 0.5305 |
| habenular commissure | hbc | 0.0% | 0.1% |  | 0.1% | 0.2% |  | 0.2598 | 0.4909 | 0.9498 |
| Other fiber tracts | Other fiber tracts | 7.9% | 6.0% |  | 10.3% | 5.5% |  | 0.5762 | 0.0950 | 0.3884 |

| Table S3N |  |  |  |  |  |  |  |  |  |  |
| --- | --- | --- | --- | --- | --- | --- | --- | --- | --- | --- |
| Brain region | Acronym | Ratio (# of cells in each class 6 region / # of cells in each class 1-5 region) | | | | |  | p value by two-way ANOVA | | |
|  |  | *Ad lib* sleep | |  | Sleep deprivation | |  | Condition | Method | Condition x Method interaction |
|  |  | Chamber | Hand |  | Chamber | Hand |  |  |  |  |
| **ventricular systems** | VS |  |  |  |  |  |  |  |  |  |
| lateral ventricle | VL | 44.7% | 33.1% |  | 42.6% | 36.7% |  | 0.9233 | 0.2890 | 0.7111 |
| subependymal zone | SEZ | 2.7% | 2.2% |  | 3.5% | 0.7% |  | 0.7536 | 0.1736 | 0.3058 |
| choroid plexus | chpl | 14.7% | 16.9% |  | 14.4% | 13.5% |  | 0.6672 | 0.8874 | 0.7223 |
| third ventricle | V3 | 20.9% | 34.3% |  | 29.4% | 27.1% |  | 0.9388 | 0.5416 | 0.4010 |
| cerebral aqueduct | AQ | 4.6% | 0.7% |  | 3.5% | 5.6% |  | 0.5453 | 0.7695 | 0.3706 |
| fourth ventricle | V4 | 4.7% | 3.2% |  | 4.7% | 0.7% |  | 0.3219 | 0.0614 | 0.2925 |
| **lateral recess** | V4r | 7.8% | 9.6% |  | 1.8% | 15.8% |  | 0.9674 | **0.0436** | 0.0852 |
| central canal spinal cord/medulla | c | 0.0% | 0.0% |  | 0.0% | 0.0% |  | - | - | - |

The ratio of cFos-TRAPed cells and two-way ANOVA results are shown for each of the 14 regions: **(A)** isocortex; **(B)** olfactory areas; **(C)** hippocampal formation; **(D)** cortical subplate; **(E)** striatum; **(F)** pallidum; **(G)** thalamus; **(H)** hypothalamus; **(I)** midbrain; **(J)** pons; **(K)** medulla; **(L)** cerebellum; **(M)** fiber tracts; and **(N)** ventricular systems. Regions with *p* <0.05 by two-way ANOVA are shown in bold.
